# The chemistry of ZnWO_4_ nanoparticle formation†
†Electronic supplementary information (ESI) available: Details regarding synthesis and characterization procedures. Tables with refinement results for different models, crystallite shapes obtained from the Rietveld refinements, additional information on the real space PDF refinements, details on the peak ratio fits, UV-VIS-DRS spectra, enlarged views of selected FFT of HR-TEM images and real space fits of the PDFs of the dry ZnWO_4_ samples. See DOI: 10.1039/c6sc01580h



**DOI:** 10.1039/c6sc01580h

**Published:** 2016-07-05

**Authors:** Espen D. Bøjesen, Kirsten M. Ø. Jensen, Christoffer Tyrsted, Aref Mamakhel, Henrik L. Andersen, Hazel Reardon, Jacques Chevalier, Ann-Christin Dippel, Bo B. Iversen

**Affiliations:** a Center for Materials Crystallography , Department of Chemistry and iNANO , Aarhus University , Langelandsgade 140 , DK-8000 , Aarhus , Denmark . Email: bo@chem.au.dk; b Department of Chemistry , University of Copenhagen , 2100 København Ø , Denmark; c Haldor Topsøe A/S , Haldor Topsøes Allé 1 , 2800 Kgs. Lyngby , Denmark; d Department of Physics and Astronomy , Aarhus University , Ny Munkegade 120 , DK-8000 Aarhus C , Denmark; e Deutsches Elektronen-Synchrotron DESY , Photon Science Division , Notkestrasse 85 , D-22607 Hamburg , Germany

## Abstract

The need for a change away from classical nucleation and growth models for the description of nanoparticle formation is highlighted. By the use of *in situ* total X-ray scattering experiments the transformation of an aqueous polyoxometalate precursor mixture to crystalline ZnWO_4_ nanoparticles under hydrothermal conditions was followed. The precursor solution is shown to consist of specific Tourné-type sandwich complexes. The formation of pristine ZnWO_4_ within seconds is understood on the basis of local restructuring and three-dimensional reordering preceding the emergence of long range order in ZnWO_4_ nanoparticles. An observed temperature dependent trend in defect concentration can be rationalized based on the proposed formation mechanism. Following nucleation the individual crystallites were found to grow into prolate morphology with elongation along the unit cell *c*-direction. Extensive electron microscopy characterization provided evidence for particle growth by oriented attachment; a notion supported by sudden particle size increases observed in the *in situ* total scattering experiments. A simple continuous hydrothermal flow method was devised to synthesize highly crystalline monoclinic zinc tungstate (ZnWO_4_) nanoparticles in large scale in less than one minute. The present results highlight the profound influence of structural similarities in local structure between reactants and final materials in determining the specific nucleation of nanostructures and thus explains the potential success of a given synthesis procedure in producing nanocrystals. It demonstrates the need for abolishing outdated nucleation models, which ignore subtle yet highly important system dependent differences in the chemistry of the forming nanocrystals.

## Abstract 2

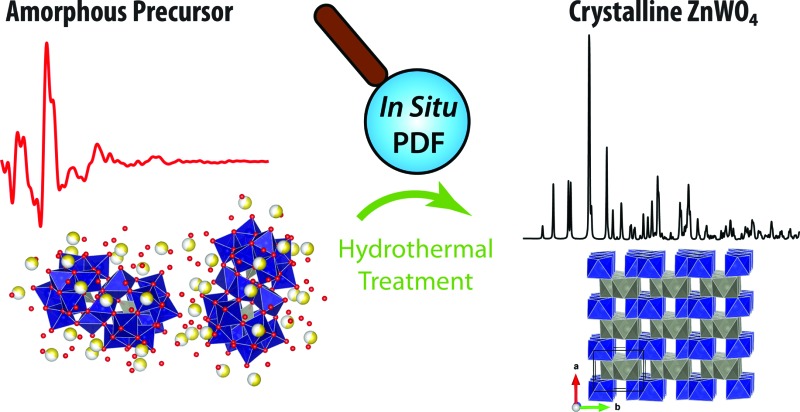
The need for a new approach to describing nanoparticle nucleation and growth different from the classical models is highlighted. In and ex situ total scattering experiments combined with additional characterization techniques are used to unravel the chemistry dictating ZnWO4 formation.

## Introduction

Chemistry is the science of matter and its changes. Nucleation of nanoparticles intuitively should be understood using the core aspects of chemistry, *i.e.* on the atomic scale where atoms are arranged in specific three dimensional geometries interacting with each other exhibiting the wide spectrum of bonding modes used in chemistry. It may thus seem strange that in the field of nanoparticle formation the actual chemistry directing the processes is most often completely ignored. Considering the importance of nanoparticle nucleation and growth not only in science, *e.g.* in the field of nanoparticle catalysis,^1^ luminescent displays and biological labels,^2^ but also in everyday life aspects, such as air quality and climate,^3,4^ it is clear that a proper atomic level understanding of the underlying chemistry controlling these processes is needed. For many years, the understanding of nanoparticle formation and growth has mainly been based on thermodynamic models that rationalize homogeneous nucleation of any crystal, including nanocrystals of complex materials, on the basis of classical nucleation theories describing any kind of phase separation occurrence bringing about an energy barrier without referral to the underlying crystal chemistry. The original model was proposed for nucleation of crystal seeds from a homogeneous mixture of molten metal by Pound and La Mer.^5^ Following nucleation, crystallite growth is often described using modified Lifshitz–Slyozov–Wagner (LSW) theory^6,7^ combined with modified Johnson–Mehl–Avrami (JMA) models^8,9^ for the description of kinetics. These original models have successfully been used to describe nucleation and growth in cases where their assumptions are valid, but increasingly in many applications of the models the assumptions are severely challenged as pointed out, *e.g.* in the review by Ludi *et al.* ^10^ for the seemingly simple oxide ZnO. In the case of complex nanosized materials synthesized by various wet chemical methods from a broad range of chemical species, the assumptions are clearly incorrect. Emerging concepts such as the existence of stable pre-nucleation structures^11,12^ and the concept of oriented attachment^13,14^ have proven successful in describing some of the lacking aspects in the classical nucleation and growth models. These concepts attempt to incorporate differences and time dependent changes in chemical bonding, surface effects, solvent–particle interactions, and dipole forces of the forming particles.^14–16^

Irrespective of model choice, the basic challenge in understanding nucleation and growth phenomena is a general lack of experimental data related to the nucleation and growth process itself, *i.e. in situ* data that relate to the nucleation event and the subsequent growth. This is especially the case when considering the processes on the fundamental atomic or chemical scale. Typical models of nucleation and growth are based on *ex situ* data, where the actual details of the nucleation and growth are inferred based on the end result. However, recently there has been tremendous progress in the application of *in situ* methods.^17–20^ Spectroscopic *in situ* methods are often the most straightforward to implement experimentally,^10,21,22^ but they only provide limited amount of direct structural information about pre-nucleation structures, in particular if these are extended in nature, as well as the long range order of the forming nanocrystals. *In situ* diffraction based methods have also been used widely^17–19,23^ to obtain valuable information on the crystalline moieties, but they cannot provide information on the amorphous structures present prior to crystallite formation. Liquid-cell *in situ* TEM studies have similarly revealed interesting features of nanoparticle formation and growth, but they are limited in the amount of sample probed, the structural information extractable from amorphous samples, reduced resolution, and on the experimental conditions achievable in the cells.^24^ A major break-through in *in situ* studies of nucleation and growth came with the application of total X-ray scattering to follow the nucleation of nanoparticles under solvothermal conditions.^25,26^ At this point a handful of such studies have been reported^17,25–30^ and they reveal an amazing diversity in the nucleation and growth of metal oxides under solvothermal conditions. In the formation of CeO_2_ nanoparticles^26^ an initial dimerization was observed, whereas for SnO_2_ and Fe_2_O_3_ the nucleation takes places through clustering of specific pre-nucleation complexes.^25,27^ The formation of ZrO_2_ nanoparticles was observed to be highly complex and occur *via* a structural breakdown of a pre-nucleation polymer chain to form an amorphous network of dimer units that eventually reorient to form the nucleus that can crystallize and grow.^17^ In the case of WO_3_ a multi-nuclear precursor cluster is observed to rearrange into a crystal structure with disorder (kinetic product).^28^ Finally, for TiO_2_ it was shown that the amorphous precursor structure has short-range order related to the anatase polymorph, and that this is decisive for determining the crystal structure of the nanoparticle.^29^ This suggests that it is not the thermodynamic equilibration which determines the TiO_2_ polymorph, but instead it is the time-dependent changes in the chemical environment.^31^ Collectively this evidence stresses that no universal model can explain the mechanisms for nucleation and growth of metal oxide nanoparticles. The chemistry and reaction mechanisms vary significantly from system to system and they are time dependent. Clearly, there is a large need for more studies providing quantitative atomic-scale structural insight into the nucleation and growth events for complex systems. Moreover, recent PDF studies on ZnO nanoparticle synthesis,^32^ solvent restructuring at nanoparticle surfaces,^33^ structural changes in metal–organic frameworks^34^ and *in operando* studies of battery materials^35^ highlight the strengths of this technique in obtaining this pivotal atomic scale information. The present study is yet another example of the strength of the total scattering approach in revealing the intricate nature of nanoparticle formation, in this case for the ZnWO_4_ system. This material was one of the first systems where oriented attachment was shown to play a vital role along with amorphous precursor particles of hitherto unknown structure,^36^ therefore an investigation into the structure of the amorphous precursor and its transformation to the crystalline is of great interest.

Here we present the first reported continuous flow hydrothermal synthesis of ZnWO_4_ nanocrystals.^37–41^ The synthesized materials are characterized using powder X-ray diffraction (PXRD), X-ray total scattering combined with pair distribution function analysis (PDF), high resolution transmission electron microscopy (HR-TEM) and scanning transmission electron microscopy coupled with energy dispersive X-ray spectroscopy analysis (STEM-EDS). In addition, ultra violet-visible infrared-diffuse reflectance spectroscopy (UV-VIS-DRS) provides a better understanding of the synthesis–structure–nanostructure–property relationships involved in ZnWO_4_. Following the *ex situ* characterization we present time-resolved total X-ray scattering coupled with PDF analysis to study the synthesis process *in situ.* In this way we (i) establish a relationship between the local structure of the amorphous precursor and the final nano-crystalline product in the hydrothermal synthesis of ZnWO_4_, (ii) explain the lack of ZnO and WO_3_ impurities when using this synthesis method, and (iii) propose a correlation between the formation mechanism and the structural properties of the final product. The study truly highlights the need for atomistic information accounting for differences in chemistry at every stage during synthesis if the goal is to obtain a real understanding of how nanoparticle systems form and grow.

## Results and discussion

### Continuous flow hydrothermal synthesis and *ex situ* powder X-ray diffraction

**Fig. 1:**
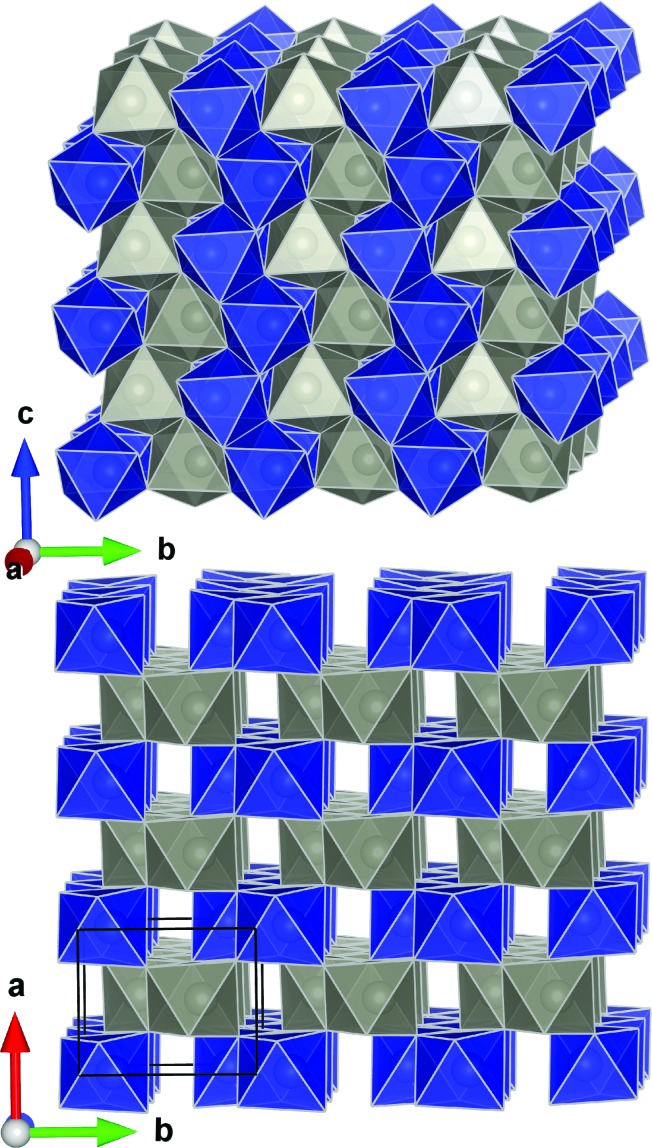
Crystal structure of ZnWO_4_ as reported by Schofield *et al.* ^49^ The WO_6_ and ZnO_6_ octahedra are shown in blue and grey, respectively.

ZnWO_4_ (zinc tungstate) has attracted attention due to its potential applications in diverse fields such as optoelectronics, as a dark matter detector material, and photocatalysis.^42–47^ It is a semiconductor with a relatively wide direct bandgap; on the order of 3.8–5.7 eV.^48^ The structure is composed of zig-zag metal–oxygen chains made up of edge-sharing ZnO_6_ and WO_6_ octahedra. Each of the (ZnO_6_–ZnO_6_)_*n*_ and (WO_6_–WO_6_)_*n*_ chains is interlinked to four chains of the other type leading to the monoclinic structure (space group *P*2/*c*) shown in Fig. 1.

All the metal–oxygen octahedra are distorted from perfect octahedral geometry. This produces three pairs of equivalent metal–oxygen (M–O) bonds in each octahedron. Covalent bonding presumably prevails in the WO_6_ units, while the ZnO_6_ units represent bonding of a mixed ionic/covalent character.^49,50^ *Ab initio* calculations suggest that the conduction band is mainly dominated by W 5d and Zn 3d states whereas the valence band has largely O 2p character.^51^ As a consequence, the electronic band structure is affected severely by changes in the local geometry of the M–O interactions. An interesting prospect for optoelectronic applications is, thus, to control the material properties in zinc tungstate by intricate control of the local M–O environment.

**Fig. 2:**
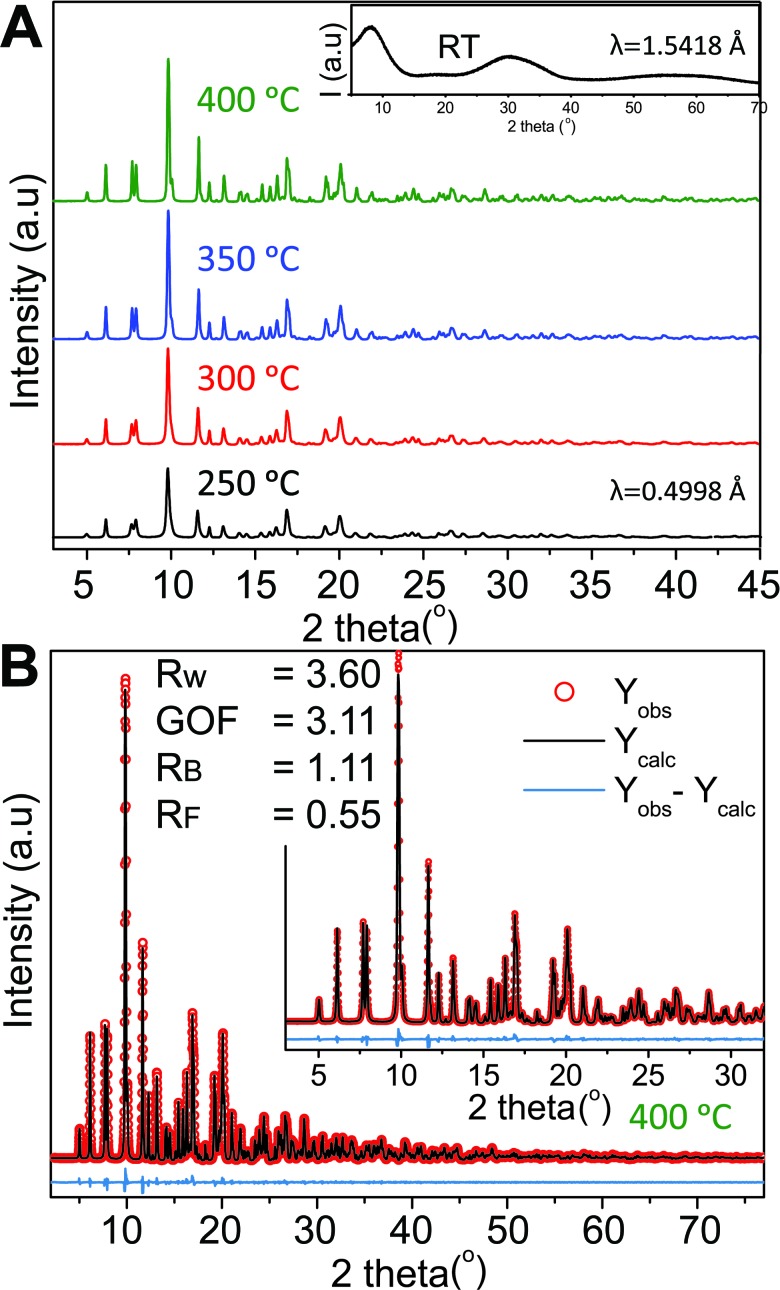
(A) High resolution synchrotron PXRD data obtained for the four different samples; the synthesis temperature is indicated next to the corresponding pattern. The inset shows the PXRD pattern of the precursor sample collected on a laboratory diffractometer at a longer wavelength than the HR-PXRD patterns. (B) Rietveld fit of the monoclinic ZnWO_4_ structure to the PXRD pattern of the sample synthesized at 400 °C. Details on the refinements are given in the Experimental section and the results are summarized in Table S1 in the ESI.†

Four samples were synthesized at different synthesis temperatures (250–400 °C) using a hydrothermal flow synthesis setup.^52^ All samples were determined to be crystalline monoclinic ZnWO_4_ based on refinements of the PXRD data (Fig. 2A and B). The initial precursor gel, vacuum-dried at room temperature, showed no distinct Bragg reflections (inset Fig. 2A) and thus is considered to be amorphous.

**Fig. 3:**
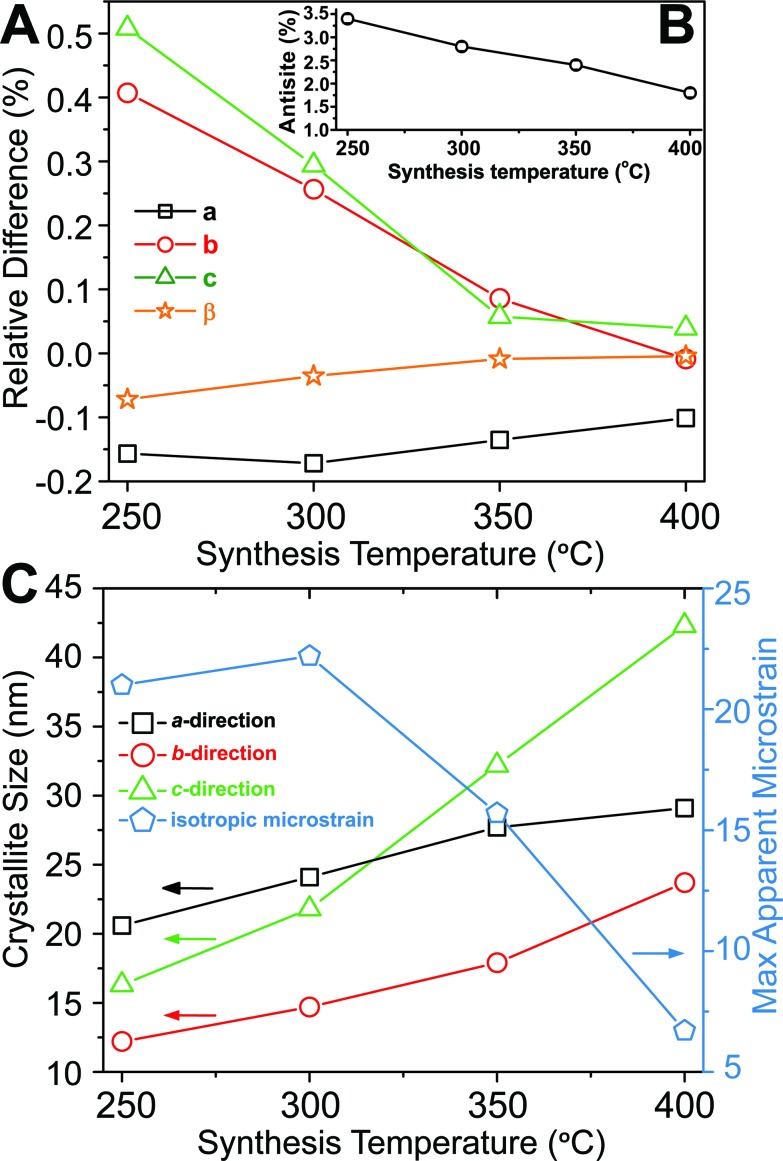
(A) Relative difference in the unit cell parameters compared with the bulk values measured at 100 K reported by Trots *et al.* ^50^ (bulk value corresponds to 0) plotted as a function of synthesis temperature. The inset (B) shows the refined anti-site defect concentration (%) as a function of synthesis temperature. (C) Crystallite sizes along the three different crystallographic directions and the isotropic microstrain (multiplied by 10 000, *i.e.*, the standard output form from Fullprof), as function of synthesis temperature. The e.s.d. for each point is smaller than the symbol sizes. The refined results can be found in Table S1.†

Based on Rietveld refinement it was found that the unit cell parameters for the monoclinic ZnWO_4_ structure were different for each sample, and Fig. 3A shows the relative difference ((refined value minus bulk value)/bulk value) in unit cell parameters as a function of synthesis temperature. The bulk values used as comparison are taken from the study by Trots *et al.* ^50^ (*a* = 4.6839(1) Å, *b* = 5.7101(1) Å, *c* = 4.9223(1) Å, *β* = 90.558(1)°). The *b* and *c* unit cell lengths decreased with increasing synthesis temperature, while the cell simultaneously expanded slightly along *a*. The monoclinic *β*-angle increased from 90.49° to 90.55°. Thus, all parameters approached the reported bulk values upon increased synthesis temperature.^50^ The combined effect of the changes in the unit cell resulted is a slight decrease of the monoclinic unit cell volume from 132.6 Å^3^ (250 °C) to 131.5 Å^3^ (400 °C). This contraction is likely correlated with differences in the nano-crystallite domain sizes and other microstructural features of the samples. The crystallite sizes were determined using a model describing the anisotropic peak broadening in the PXRD pattern with spherical harmonical functions as described by Popa^53^ and Jarvinen^54^ and implemented in Fullprof.^55^ The largest changes in the unit cell parameters occurred along the same directions as where the largest changes in crystallite size were observed, *i.e. c* and *b* (Fig. 3C).

To obtain a proper fit of the PXRD patterns it was necessary to introduce defects into the structural model. Several models were tested (see Experimental section and ESI for details†) and the best compromise between quality of fit, retention of mass- and charge balance, and number of refined parameters was achieved by incorporating an anti-site model, where a fraction of the Zn and W atoms switch positions. Other defects such as site-vacancies may also be present, but here we choose to simplify the model to avoid over-parameterization. Fig. 3B shows the refined concentration of anti-site defects in the four different samples. The reported concentrations are defined as the amount of Zn on a W site (and conversely W on a Zn site). Based on the refined values it appears that an increased synthesis temperature leads to lower concentrations of anti-site defects, and similarly the isotropic atomic displacement parameters for both Zn and W were found to decrease as a function of synthesis temperature (see ESI†). Overall, the results demonstrate the presence of temperature dependent static disorder in the structure. PDF analysis of total scattering data on the same samples (ESI†) revealed no significant amorphous contributions or local structural distortions beyond the monoclinic *P*2/*c* structure.

**Fig. 4:**
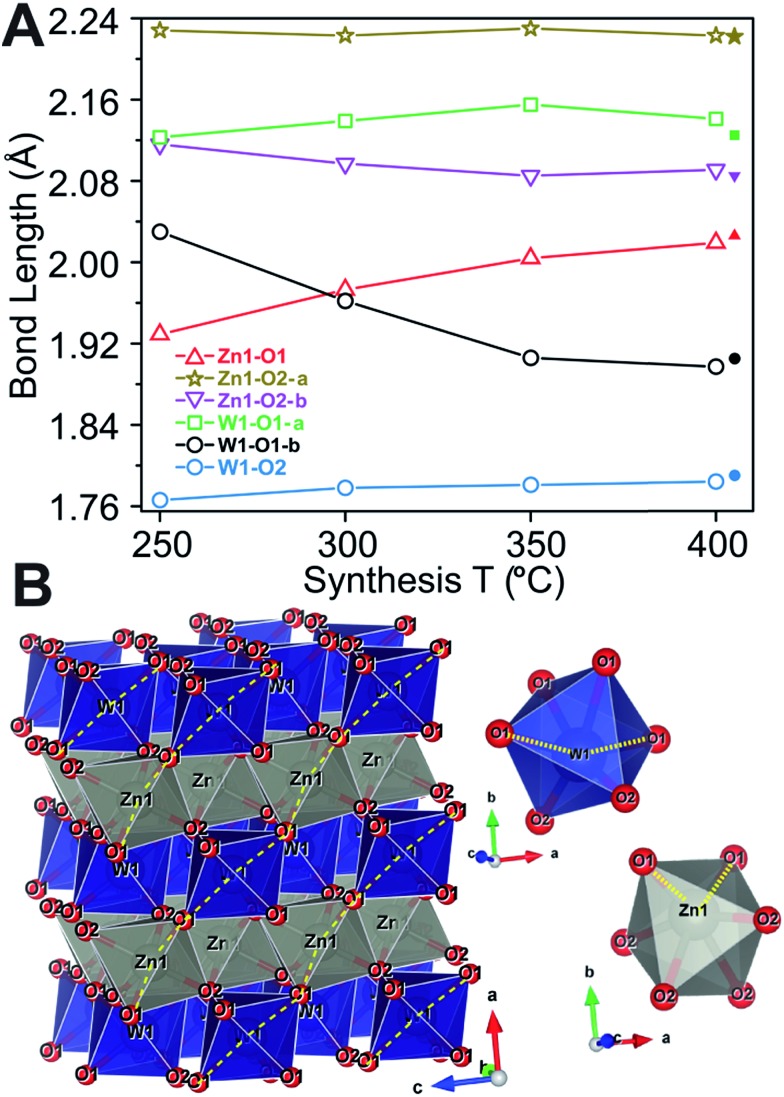
(A) M–O bond lengths as a function of synthesis temperature, where filled symbols on the right hand side of the diagram indicate the bulk values (B) illustration showing the different M–O bonds in the ZnO_6_ (grey) and WO_6_ (blue) octahedra, respectively. The dashed yellow lines indicate the bonds determined to exhibit the strongest temperature dependency, *i.e.* W1–O1-*b* and Zn1–O1.

Peak-profile analysis of the PXRD patterns revealed that the volume-averaged crystallite domain sizes generally increase with increasing synthesis temperature. The change in crystallite size was different along all three crystallographic directions. The refined anisotropic crystallite sizes are plotted in Fig. 3C showing that growth along *c* is significantly more pronounced than along *a* and *b*, leading to a prolate crystallite morphology for the highest synthesis temperatures (an example of the refined prolate morphology is depicted in the ESI†). The apparent maximal isotropic microstrain was also determined from peak profile analysis, and it decreases with increasing synthesis temperature (Fig. 3C, secondary *Y* axis). This trend supports the presence of high concentrations of defects in the samples synthesized at low temperatures. Significant changes in M–O bond lengths were observed as a function of synthesis temperature (Fig. 4A), where bonds involving the O1 oxygen atoms were found to be particularly affected (Fig. 4B). This reflects the influence of the relatively large differences in crystallite size along the *c*-direction for the different samples. The samples synthesized at lowest temperature exhibited M–O bond lengths which differed significantly from the reported bulk values. However, the extracted M–O bond lengths approached the reported bulk values upon increasing synthesis temperature.

UV-VIS diffuse reflectance spectra were used to estimate the direct band gap values for the four different ZnWO_4_ samples and they were found to be 3.8–4.1 eV. The band gap decreased with increasing synthesis temperature (see ESI†). This trend is most likely related to the observed differences in bond length and anti-site defect concentration. Furthermore, several features in the UV-VIS data may be attributed to presence of various defects or dynamic disorder. Previous reports have found that the degree of distortion in WO_6_ structures will influence the band gap significantly.^56^ Thus, it appears that it is possible, to some degree, to control the nature of the band gap and accordingly the optical and photo-catalytic properties of ZnWO_4_ by control of the synthesis temperature.

### Nanostructure and elemental distribution from electron microscopy

**Fig. 5:**
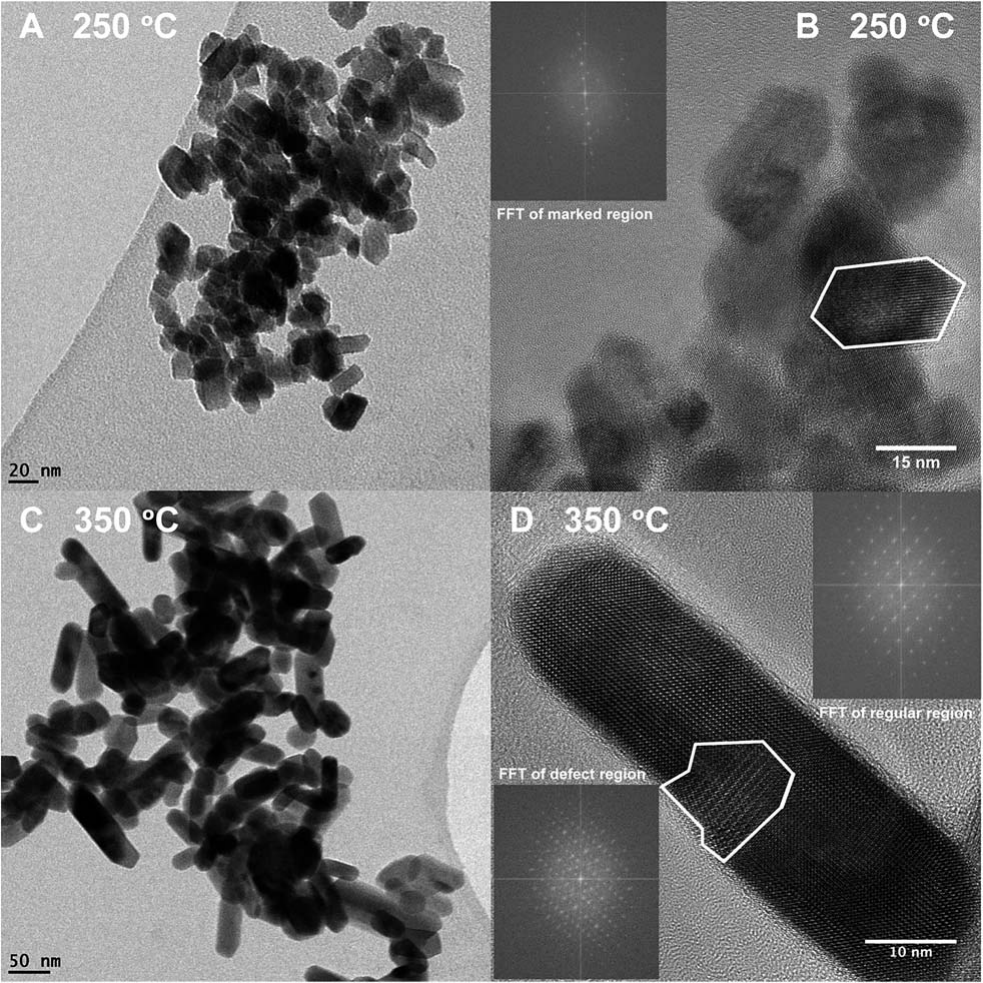
(A) TEM image of a sample synthesized at 250 °C (B) HR-TEM image of a sample synthesized at 250 °C; inset is a FFT of the outlined particle. (C) TEM image of a sample synthesized at 350 °C. (D) HR-TEM image of a sample synthesized at 350 °C; the inset at the upper right is a FFT of an arbitrarily selected region containing no defects. The inset in the lower left corner is the FFT of the region outlined in white, where additional spots can be seen in the latter inset in comparison to the former inset (enlarged views of the FFT are placed in the ESI†).

The TEM pictures in Fig. 5 illustrate the nanorod morphology of the particles produced by the present hydrothermal flow method, and they support the trends obtained from the Rietveld refinements, *i.e.* increasing synthesis temperature results in larger, more anisotropic particles. Similar morphologies have previously been reported for ZnWO_4_ synthesized by batch hydrothermal synthesis and the growth of these types of nanorods has been suggested to occur *via* oriented attachment growth mechanisms.^36,57^ The particle size distribution broadens (Fig. 5A *vs.* C) with increasing temperature, and additionally some of the particles synthesized at the highest temperature are significantly larger than the largest sizes extracted from the PXRD analysis. This reflects that the large particles not necessarily are single crystals. In the HR-TEM image of the smallest observed particles, *i.e.* those obtained at a synthesis temperature of 250 °C, it can be seen that the ZnWO_4_ particles are highly crystalline. Fast Fourier transform (FFT) of the region outlined in Fig. 5B reveals discrete ordered spots, indicating the crystalline nature of the particle. However, it is pertinent to note that TEM and STEM images probe local regions, and thus amorphous particles may still exist within the bulk product matrix. Nevertheless, it is interesting to discover that the particles produced by the fast flow synthesis method appear to be highly crystalline, despite the very short reaction time compared with batch autoclave syntheses.

Fig. 5D shows a HR-TEM image of a particle synthesized at 350 °C. The area outlined in white reveals defects within one ZnWO_4_ particle, or possibly an entire crystallite oriented in a different manner than the rest of the particle. Simply by direct visual inspection of the image, this defect region can clearly be distinguished from the regularly ordered arrangement surrounding it. The FFT of the outlined defect region in Fig. 5D (inset in bottom left corner) contains additional spots in comparison to the FFT of the defect-free region of the particle, providing clear evidence of the different structure or orientation of this particular region. Combining the observations from TEM and HR-TEM with the refined values of microstrain and anti-site concentrations from the PXRD data indicates that a relatively large number of defects can be accommodated within the small particles. Moreover, these defects were found to influence the local M–O environment leading to increased distortion of the MO_6_ octahedra (Fig. 4A) and thus possibly the opto-electronic properties.

**Fig. 6:**
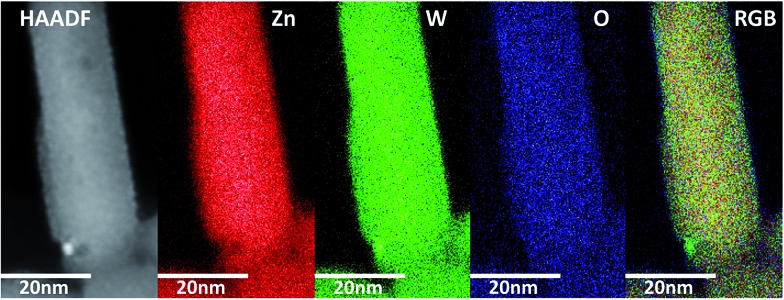
From left to right: STEM-HAADF image of ZnWO_4_ nanocrystal with a small impurity/inclusion. Elemental maps of the distribution of Zn, W, and O respectively. At the far right a RGB composite image of the three elemental maps is shown, highlighting the W rich nature of the small impurity.

STEM-EDS experiments were performed in order to investigate whether the elemental distribution was homogenous throughout the nanoparticles. Small amounts of minute tungsten rich inclusions were detected in some of the synthesized products (Fig. 6). Furthermore, STEM micrographs acquired using a high-angle annular dark field detector (HAADF) revealed that the nanorods have buckled surfaces, *i.e.* not atomically sharp facets. This aspect will be discussed in greater detail later.

**Fig. 7:**
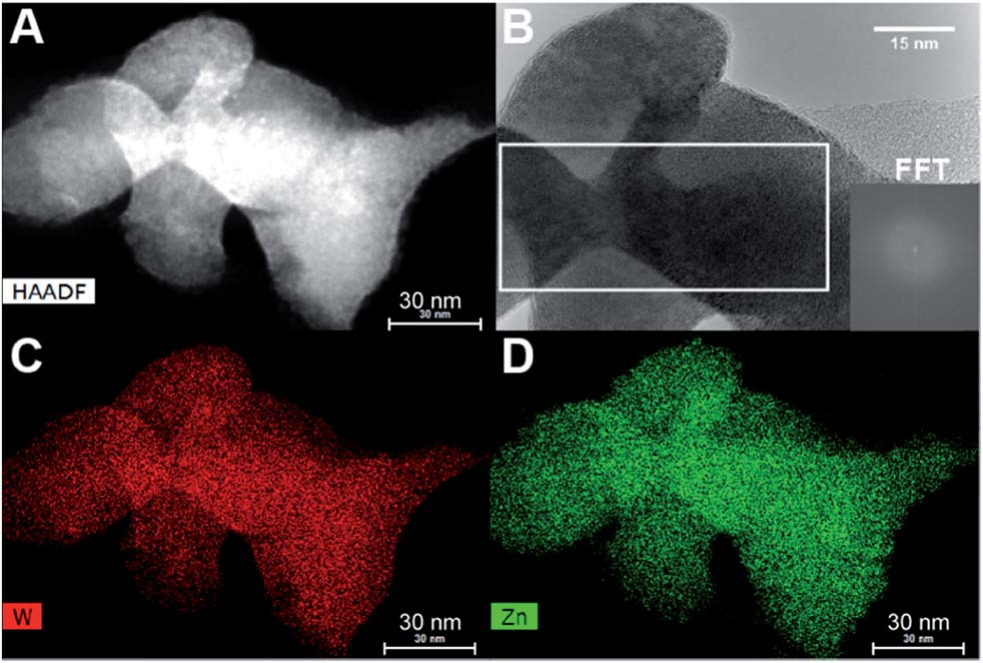
(A) STEM-HAADF picture of a RT-precursor sample. (B) HR-TEM picture of the same sample; inset shows FFT of the outlined region, where two minute spots can be seen. The elemental maps of the same area of sample are given in (C) and (D), which demonstrate the homogeneous distribution of W and Zn.

STEM-EDS mapping of the dried precursor sample was also performed and the resultant elemental maps shown in Fig. 7 reveal a homogeneous distribution of Zn and W. Additionally, HR-TEM imaging (Fig. 7B) confirmed that the precursor structure is indeed predominantly amorphous with no evidence of lattice fringes. Close inspection of this image revealed the presence of very few nanocrystallites, although in such minute concentrations that their resultant Bragg peaks could not be detected in the PXRD data of the dried precursor. The homogeneous distribution of elements thus points towards a precursor consisting of a single amorphous phase and not a heterogeneous composite of various types of poorly mixed compounds or components.

### Precursor structure from *ex situ* X-ray total scattering

**Fig. 8:**
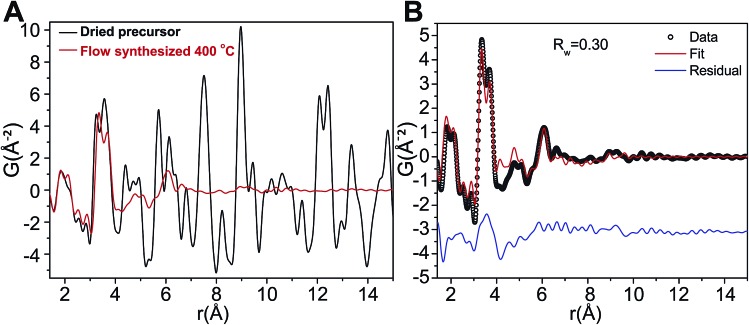
(A) PDF of the dried precursor sample (black) and of the dried 400 °C flow synthesized sample (red). (B) Fit of the dry precursor data based on a distorted version of the Na_12_[WZn_3_(H_2_O)_2_(ZnW_9_O_34_)_2_] structure model and using the program PDFgui with a size damping envelope.^58^

The relatively low temperatures and short reaction times needed for production of ZnWO_4_ nanocrystals may be understood by investigating the precursor used in greater detail. Based on Fig. 7 it is clear that the precursor possesses a homogeneous distribution of elements. To further investigate the precursor, the local atomic structure of the dried precursor mixture was probed by PDF analysis to enable a thorough investigation of the material prior to reaction. Fig. 8A shows the low *r*-region of the PDFs of both the dried precursor and the sample synthesized at 400 °C. Definite similarities between the local structure of the precursor and the final crystalline product do exist, as can be clearly seen upon comparison of the profiles.

**Fig. 9:**
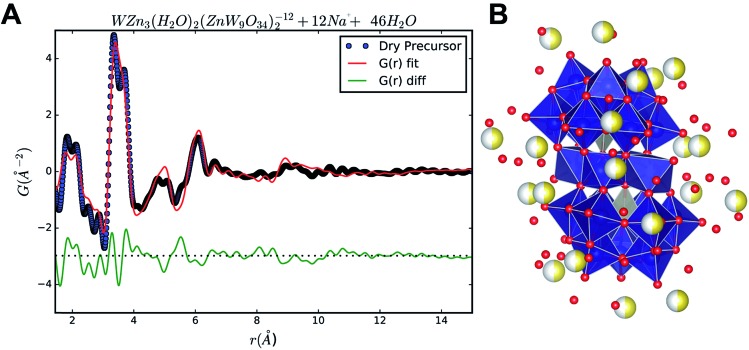
(A) Fit of the precursor PDF using the Debye equation and the program Diffpy-CMI.^60^ (B) The cluster used in the refinement, based on the result from the PDFgui refinement. Blue atoms are W, grey are Zn, yellow are Na and red are O. The sodium sites are all only 50% occupied, and the *R*_w_ value was 0.31.

**Fig. 10:**
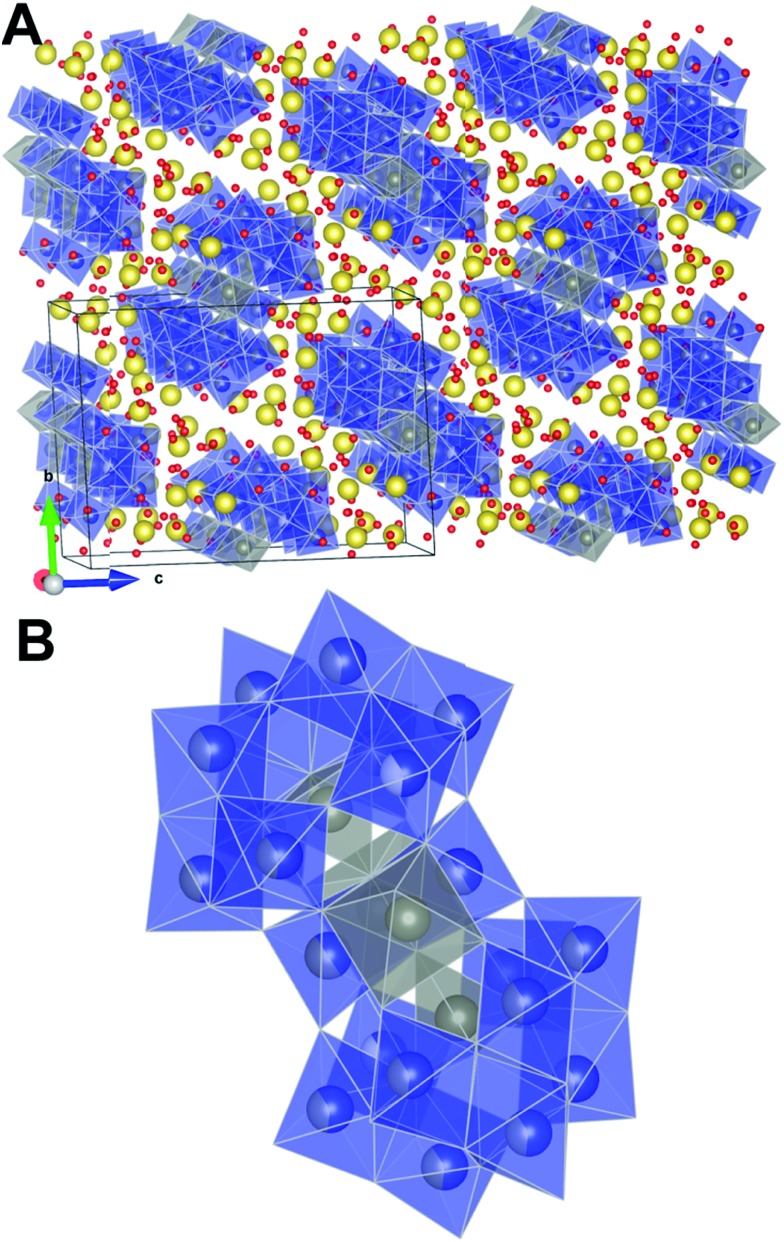
(A) The parent Na_12_[WZn_3_(H_2_O)_2_(ZnW_9_O_34_)_2_] with its main structural moiety the disordered Tourné sandwich ion shown in (B); the distorted Tourné ion, the Zn- and W-centered polyhedra are grey and blue, respectively, yellow spheres are Na atoms while red spheres represent oxygen from water molecules. Oxygen at the corners of the polyhedra are not shown. Here the disordered version with mixed occupancies is shown; these are represented by the fractional coloration of the spheres at the respective positions.

The PDF of the precursor could be fitted by a model derived from the polyoxometalate^59^ Na_12_[WZn_3_(H_2_O)_2_(ZnW_9_O_34_)_2_] (Fig. 10A)^58^ with a refined spherical particle diameter of 11.5 Å. The fit is shown in Fig. 8B, and a graphical representation of the extended structure is shown in Fig. 10. There are minor discrepancies in the fit, but the overall agreement between the model and data is good (*R*_w_ = 0.30). A 1 : 1 ratio of Zn and W was used for precursor preparation, however, the original Na_12_[WZn_3_(H_2_O)_2_(ZnW_9_O_34_)_2_] structure is Zn deficient compared with the 1 : 1 mixture employed here. It is expected that the excess Zn in the precursor mixture occupies sites otherwise occupied by W in the reported structure. Thus, to accommodate a Zn : W ratio of 1 : 1 in the PDF refinements of the precursor model the occupancies of the octahedral sites in the top and bottom part of the Tourné sandwich ion were to 0.6125 W and 0.3875 Zn, respectively, Fig. 10B. On average this produced an assumed Na_12_[WZn_3_(H_2_O)_2_(ZnW_5.5125_Zn_3.4875_O_34_)_2_]^16–^ composition that described the precursor appropriately. This change did not produce a significant change in the quality of the fit. The unbalanced charge is assumed to be counterbalanced by some of the coordinating oxygen being OH^–^, predominately in the Zn octahedra. The chosen stoichiometry gives an approximate 1 : 1 : 1 ratio of the cations from which the precursor mixture was prepared. The coordinates of the atoms in the refined precursor model differ from the ones in the reported structure. This is most likely due to the actual precursor consisting of a broad range of clusters with structural similarities. One possible scenario is an ensemble of a multitude of distorted “Tourné sandwich ion”-like structures. In particular a larger degree of edge sharing octahedra are present in the refined structure compared with the original structure. To verify that the good fit in PDFgui was not an effect of using a program designed for periodic structures on an amorphous compound, additional refinements were prepared using the program Diffpy-CMI.^60^ In these refinements the model PDF was calculated using the Debye-equation. The input structure was a cut-out from the structure obtained from the PDFgui refinements. One single Tourné sandwich ion surrounded by 24 Na sodium sites (only 50% occupied to produce charge balance) along with additional 46 water molecules make up the suggested model. The atomic positions were kept fixed to the values obtained from the PDFgui model and the only parameters, which were refined, were isotropic thermal parameters, a scale factor, a factor for correlated motion and finally an overall factor used for expanding and contracting the entire assembly. The result of this fitting is shown in Fig. 9 alongside the cluster itself, and the coordinates and B values can be found in a table in the ESI.† In this fit the originally reported Zn : W ratio was kept, but a fit using a 1 : 1 ratio produced fits of similar quality.

The obtained fit was reasonable *i.e.*, *R*_w_ 0.31, but produced relatively large atomic displacement factors. In the model the double peak at approx. 4 Å is lacking some “fine structure”. This feature is most likely a manifestation of forcing all atoms of one type to have the same ADPs. In reality the short range order seems rather distinct whereas the signal for correlations above 5 Å reveals increasing amounts of disorder. Combining this observation with the fact that the fit in PDFgui employs a size dependent dampening envelope on the intensities provides additional indications about the nature of the precursor structure. This, however, requires a closer look at the main structural motif in the parent structure used *i.e.* the so-called Tourné type sandwich ion, depicted in Fig. 10B. The ion consists of two connected fragments of the Keggin-type poly-anion, which are well known structures in polyoxometalate chemistry.^59,61,62^ In each Keggin-type fragment three edge-sharing octahedra are missing, effectively producing [ZnW_9_O_34_]^12–^ units, assuming the reported stoichiometry. Two such units are linked together by a layer of four close-packed octahedrally coordinated metal atoms. The intermediate layer consists of sites with different symmetries, where Zn and W each occupy two of the sites in a random manner. Fig. 10 shows disordered Zn and W mixed in both halves of the sandwich ion used for refinements in PDFgui, highlighted by the mixed coloration of the atoms. In this poly-oxo-tungstate ion not all metal ions are octahedrally coordinated; in each half of the sandwich one tetrahedrally coordinated Zn is “cradled” by a number of octahedrally coordinated metal atoms.

Considering these structural features and the two different approaches for fitting its PDF it thus appears likely that the precursor structure has many features in common with the Tourné sandwich ion but with a large degree of disorder, in particular at the top and bottom ends of the ion. It is very likely that an ensemble of differently distributed connections and orientations of the individual octahedra exits, whereas, the layer of close packed octahedra appears to act as a quite rigid unit present in many clusters.

The formation of the proposed structure in the precursor mixture agrees well with existing literature. Samples prepared by a synthesis method similar to that used here (*i.e.*, by co-precipitation of aqueous solutions of ZnSO_4_ and Na_2_WO_4_) were studied by Kalinko *et al.* ^63^ using extended X-ray absorption fine structure (EXAFS) analysis. They reported that the average coordination of O to Zn in the precipitated powders was lower than six. A purely octahedral coordination of Zn as found in ZnWO_4_ should produce an EXAFS signal indicative of six-fold coordination. Kalinko *et al.* ^63^ assumed that the nanocrystallites of ZnWO_4_ formed from their reaction mixture were less than 2 nm. This assumption was based on the absence of Bragg peaks in the PXRD patterns of the samples, *i.e.* it is stated that the phase is indeed ZnWO_4_, but that the crystallite size is too small to produce distinct Bragg peaks. An alternative explanation is that the precipitates investigated by Kalinko *et al.* ^63^ were actually amorphous nanoparticles akin to the ones found in our study, and not ZnWO_4_ nanocrystallites. Tourné-sandwich ions produce an average Zn coordination number between 5.3 and 5.7 (depending on the assumed Zn/W disorder on the sites usually occupied by W), and Kalinko *et al.* reported an average Zn coordination number in this range. Thus, the interpretation of the EXAFS data collected by Kalinko *et al.* ^63^ overlooks the evidence to suggest that Tourné sandwich ions or structures akin to it exist in the precursor. The correlations in the precursor PDF were observed to about 11 Å. This distance coincides with the longest M–M correlations found in the Tourné type sandwich ions and supports the presence of these 1 nm sized clusters. Fits of the precursor PDF using nanosized ZnWO_4_ gave poor fits (*R*_w_ > 50%) further supporting the presence of a differently structured precursor moiety. Fits using simple Keggin clusters also gave poor fits and it led to unphysically short M–O distances (see ESI†) and simulations based on the Debye equation also showed that Keggin clusters do not produce detectable features above 8 Å and therefore cannot explain the clear broad features above 8 Å in the experimental PDFs.

Remarkably, Tourné *et al.* ^58^ observed that only by mixing stoichiometric amounts of the different components in a controlled manner it is possible to produce pristine crystalline Na_12_[WZn_3_(H_2_O)_2_(ZnW_9_O_34_)_2_]. Rapid mixing or preparation of non-stoichiometric mixtures led to amorphous products. This observation, albeit based on a slightly different synthesis mixture, serves to explain that crystalline Na_12_[WZn_3_(H_2_O)_2_(ZnW_9_O_34_)_2_] does not form in the precursor mixture in the present study. The small W-rich impurities detected by STEM-EDS in the final product (Fig. 6) could be crystalline precipitates of this poly-oxo-tungstometalate. To the best of our knowledge, the electronic band structure of Na_12_[WZn_3_(H_2_O)_2_(ZnW_9_O_34_)_2_] is not known. The band gap of the precursor sample was extracted under the assumption of a direct band gap and gave a value of 4.55 eV and this is in accordance with suggested values for small poly-oxo-tungstate clusters containing *ca.* 6–9 distorted and octahedrally coordinated W–O polyhedra,^56^ and therefore also in agreement with the suggested precursor model.

### The chemistry of nucleation from *in situ* X-ray total scattering

The relationship between the precursor structure and the final product was investigated in greater detail by the use of *in situ* total scattering combined with PDF analysis. Together with the refinements on the dry precursor, this allows us to suggest a formation mechanism for the crystalline structure. A comparison between the PDF of the dry and the “wet” precursor showed almost the same structure, albeit with a difference in the double peak describing the first metal–metal distances, indicating a larger degree of corner sharing and overall order upon drying (see ESI†).

**Fig. 11:**
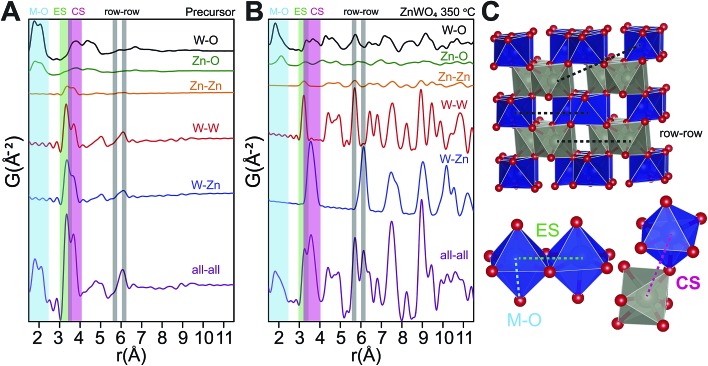
(A) Partial and total calculated PDFs of the refined precursor structure model (using PDFgui). (B) Partial and total calculated PDF of the refined ZnWO_4_ model based on the fit to the 350 °C sample (see ESI†). (C) Illustration of some important distances: row–row metal–metal correlations (*r*–*r*), edge sharing metal–metal correlations (ES) and corner sharing metal–metal correlations (CS). The row–row correlations clearly only exist in the final product.

To identify the interatomic distances characterizing the various structural motifs in the precursor, the calculated partial PDFs of the refined precursor structure (Fig. 11A) and the refined ZnWO_4_ model of the 350 °C synthesized sample (Fig. 11B) are first considered. The partial PDFs show that in the low *r*-region between 1.5 and 2.5 Å, the peaks found in both structures arise due to metal–oxygen bonds, which involve the octahedrally coordinated Zn (Zn_O_) and W (W_O_) for both structures, and tetrahedrally coordinated Zn (Zn_T_) in the case of the precursor structure.

The shortest metal–metal (M–M) distances are found in the 3–4 Å region (Fig. 11). The doublet present in both structures reflects the two main types of nearest M–M distances: edge-sharing (ES) octahedra (shorter distance) and corner sharing (CS) octahedra (longer distance). ES octahedra make up a relatively large proportion of the octahedra found in the “Tourné sandwich-like” ion whereas in the ZnWO_4_ structure only octahedra with the same type of metal center exhibit ES. CS between octahedra parallels this behavior, where CS occurs for all types of octahedra in the precursor structure, while it is limited to purely W–W or Zn–Zn distances in the final product. The differences observed in the shortest M–M peak ratios for the two structures (Fig. 11A *vs.* B) are thus readily explained by these structural disparities and can be used to assess the similarity of a given dataset with one structure or the other. Finally, at *ca.* 6.1 Å in the sandwich-type structure (Fig. 10 and 11A) a broad peak is observed due to a large number of very similar next nearest M–M distances. Conversely, in the crystalline ZnWO_4_ nanoparticles (Fig. 11B and C), each type of M–M correlation in this region (5.9–6.2 Å) is centered on slightly different distances. The distinct distance splits are caused by the “row–row” distances in the final structure, a structural feature which does not exist in the precursor and thus is a clear indicator of the presence of the ZnWO_4_ structure.

### Formation and growth mechanism

**Fig. 12:**
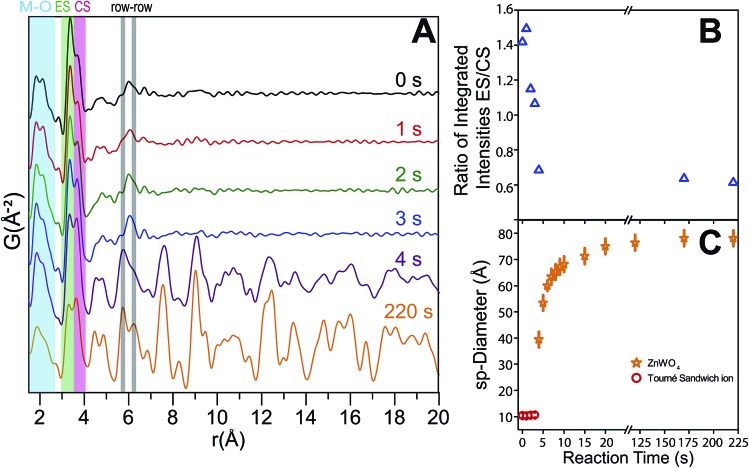
(A) Low *r* region of PDFs for the reaction as a function of time. The important CS, ES and, *r*–*r* distances are indicated. (B) The ratio between the integrated intensities of the CS and ES peak, *i.e.*, CS/ES as a function of synthesis time. A large change in this ratio is observed up to 4 s of reaction, which is the point in the reaction where the first ZnWO_4_ particles are seen to form. (C) The refined sp-diameter as a function of reaction time. This parameter is related to the particle size; however, the anisotropic shape of the particles prohibits a fully quantitative comparison of the sizes.

The time-resolved PDF of the precursor mixture in solution (Fig. 12A) is very similar to the PDF of the dried precursor sample. Immediately after heating is initiated, restructuring of the precursor takes place. Within the first three seconds of heating, changes in the local M–O environment occur, indicated by the slight change in the intensity ratio of the corresponding peaks in the 1.5–2.5 Å region (Fig. 12A). The origin of these changes is believed to be the conversion of Zn_T_ to Zn_O_, as well as accompanying changes in local geometry and connectivity of the M–O octahedra. Simultaneously, the nature and distribution of M–M correlations also changes; the intensity of the CS peak is intensified while the ES peak is diminished. This trend is illustrated in Fig. 12B where the ratio between the integrated intensities of the ES and CS peaks (fitted with a double Gaussian function, see ESI†) is shown as a function of reaction time. The trend can be understood by considering the initial multimodal distribution of closest W–W, Zn–Zn, and W–Zn distances caused by a mixture of ES and CS octahedra present in the precursor structure, which quickly transform to purely ES arrangements for W–W and Zn–Zn and purely CS Zn–W in the final pristine ZnWO_4_. It is noteworthy that the particle radius did not appear to change during the first three seconds, *i.e.*, correlations to about 11 Å were present at all times. This observation indicates that a rearrangement, without complete fragmentation is taking place. After four seconds of reaction (Fig. 12A) the broad peak with its maximum centered around 6.1 Å rises in intensity and clearly splits into two distinct maxima. The appearance of these maxima, caused by the formation of row–row correlations, indicates the onset of ordering into zig-zag rows of ES octahedra connected *via* CS to four other rows. Simultaneously, correlations up to the *r* range of several nanometers appear. The nanometer sized particles formed at this point already present structural characteristics which highly resemble the final pristine ZnWO_4_.

The ratio of ES to CS M–M correlations after 4 s has almost, but not entirely, reached its final value (*i.e.* the value after 220 s). The difference between the ratio after 4 s and 220 s can be explained by a transformation from an initially highly disordered ZnWO_4_ (*i.e.* containing a high concentration of anti-site defects with Zn occupying W positions and *vice versa*), to a fully ordered ZnWO_4_ structure. This interpretation can also explain the trend of decreasing anti-site defect concentration and microstrain with increasing synthesis temperature as suggested from the *ex situ* PXRD refinements. The 4 s data required a mixture of both the precursor phase (using the result from the dry precursor fit refining only the sp-diameter and scale) and the final ZnWO_4_ phase to produce proper fits in PDFgui. The sp-diameter of the precursor phase after 4 s was actually found to decrease to around 8 Å, and this may be an indication of some restructuring of the sandwich ions or simply an artifact of having a two phase mixture at this state. It is pertinent to note that the actual sizes are not directly related to the crystallite sizes determined by PXRD due to the anisotropic shape of the particles.

**Fig. 13:**
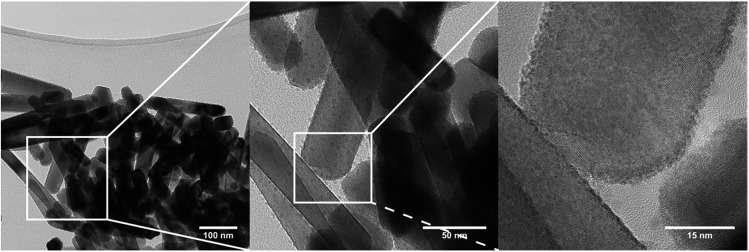
TEM micrographs of flow synthesized ZnWO_4_ samples. Smaller particles aggregate into larger ones in an oriented fashion. No elemental inhomogeneity was found to exist between the small particles and large rods (based on STEM-EDS).

The sudden increase in particle size (sp-diameter, Fig. 12C) may be understood on the basis of oriented attachment occurring during the growth; a mechanism which previously has been shown to be prevalent during solution based synthesis methods in this compound.^36^ In this model small particles, possibly not entirely transformed to ZnWO_4_ yet, attach to each other in an oriented way due to growth-directing forces, such as, coulomb interactions and van der Waals interactions. The presence of the defects observed in the PXRD study and HR-TEM images is supporting this suggestion. Furthermore, in the TEM micrographs shown in Fig. 13 (and STEM HAADF picture in Fig. 6) it can be seen that the large rod-shaped particles at the surface appear to consist of a large number of mutually aligned smaller particles.

The *in situ* total scattering study thus reveals important information about the limiting steps in the formation of ZnWO_4_. An initial change in coordination and connectivity between the M–O polyhedra in the precursor is necessary before it becomes energetically favorable for the connected octahedra to order into the zig-zag rows, thus defining the wolframite structure. Interestingly, this postulate holds true even if the suggested “Tourné sandwich-like” precursor structure is not correct, since the distinct M–O and M–M distances which are followed in the *in situ* study (corner *vs.* edge sharing) are present in many different polyoxometalate clusters. No evidence was found to suggest a breakdown into smaller units, as has previously been reported for the formation of Y-stabilized ZrO_2_.^17^ It is somewhat surprising that ZnO is not observed at any point during the synthesis. The synthesis conditions (pH *ca.* 7 and temperatures ≥ 250 °C) are close to optimal for ZnO formation according to literature.^23,39^ The lack of ZnO, which has the wurtzite structure with complete tetrahedral Zn coordination, may be explained by the stability and resemblance in local structure of the Tourné ion to the ZnWO_4_ structure. The presence of octahedrally coordinated Zn and mixture of Zn–W is a crucial factor that facilitates the formation of pristine ZnWO_4_ based on a precursor mixture of aqueous solutions of Zn(NO_3_)_2_ and Na_2_WO_4_. This observation fits in with the emerging understanding that the local structure of precursors play a vital role in directing structural evolution in various systems.^64^

## Conclusion

Monoclinic ZnWO_4_ nanocrystallites were produced at four different temperatures (250 °C, 300 °C, 350 °C and 400 °C) by a continuous flow hydrothermal synthesis method. The individual crystallites were found to be of prolate morphology, elongated along the *c*-unit cell direction. The average crystallite dimensions and aspect ratio increased with increasing synthesis temperature, which also lead to a decreasing microstrain and anti-site defect concentration. The band gap energies of the nanocrystallites were 3.8–4.1 eV.

X-ray total scattering experiments revealed the structure of the amorphous precursor to be consisting of some type of poly-oxo-tungstate anions (with a structure resembling distorted Tourné type sandwich ions). *In situ* total scattering studies and PDF analysis revealed that local restructuring and three-dimensional reordering of the Tourné type sandwich ions precursor precedes the emergence of long range order during the hydrothermal synthesis of ZnWO_4_. The results highlight the profound influence of structural similarities in local structure between precursors and final compounds in determining the course of a given material formation. It is the octahedral Zn coordination and structural similarity between the precursor and ZnWO_4_ that *e.g.* prevents ZnO (tetrahedral Zn coordination) to be formed. The suggested nucleation mechanism also explains why highly crystalline and phase pure ZnWO_4_ nanoparticles can be synthesized in less than one minute at 250 °C in a continuous flow hydrothermal reactor. This is in strong contrast to a reaction time of 24 h at 1000 °C used in conventional solid state synthesis based on ZnO and WO_3_ reagents.^65^

The present study is thus a firm testament to the necessity of a paradigm shift in the view on the nucleation of nanoparticles. Advances in synchrotron sources, software, and experimental setups open up the possibilities for moving away from simple unified descriptions which disregard subtle, yet, extremely important differences in the chemistry of the various nanoparticle systems. In molecular chemistry delicate bond rearrangements and the specific nature of chemical interactions have long been used to explain chemical phenomena. It is time these core concepts of chemistry are also used in the chemical reactions of solid state science. In the present case of ZnWO_4_ an intricate control of precursor preparation is of critical importance when optimizing nanoparticle syntheses and it presents an interesting handle for delicate synthesis control and rational manipulation of the nanoparticle properties.

## Methods

### Flow synthesis

For all syntheses, Na_2_WO_4_·2H_2_O (99%, Sigma-Aldrich) and Zn(NO_3_)_3_·6H_2_O (≥99%, Sigma-Aldrich) were used as received. Equimolar amounts of W and Zn precursors were dissolved in deionized water in two separate containers leading to transparent, colorless solutions. Upon mixing, a white precursor gel was formed instantaneously. The final metal ion concentration in the precursor was calculated to be [Zn^2+^] = [W^6+^] = 0.05 M. Syntheses were performed at four different temperatures using a T-piece hydrothermal flow apparatus described previously (additional information in the ESI†).^52,66,67^

### Powder X-ray diffraction and Rietveld refinements

The prepared powders were studied by high resolution synchrotron powder X-ray diffraction (PXRD) at beamline BL44B2 at the SPring8 synchrotron in Japan (details in ESI†). The powder patterns were analyzed by Rietveld refinement to extract structural and microstructural information using the *Fullprof* software suite,^55^ where refinements were based on the monoclinic crystal structure of ZnWO_4_ as reported by Schofield *et al.* ^49^ Details regarding the refinements, *e.g.* the profile model used and details of the antisite model are given in the ESI.†

#### *Ex situ* X-ray total scattering

*Ex situ* X-ray total scattering measurements on the dry samples were performed at beamline ID11 ESRF, Grenoble, France with a *Q*_max_ of 21 Å^–1^.^68^ The experimental *Q*_damp_ was determined to be 0.0215 Å^–1^ by refinement in PDFgui^69^ of data from a LaB_6_-calibrant measurement. X-ray total scattering measurements on the dried and washed precursor were performed at beamline P02.1, PETRA III, DESY, Hamburg, Germany.^70^ This setup produced a usable *Q*_max_ of 18 Å^–1^ and the experimental *Q*_damp_ was found to be 0.02246 Å^–1^. In all cases (including the *in situ* data below) the data were integrated using *Fit2D*.^71^ Total scattering pair distribution functions (PDFs) were obtained through PDFgetX3,^72^ while real space Rietveld refinements were performed in *PDFgui*.^69^ Additional PDF refinements using the Debye scattering function were carried out in the program Diffpy-CMI^60^ using the experimental *Q*_min_, *Q*_max_, and *Q*_damp_ and only refining isotropic thermal parameters, scale factor, a factor for correlated motion and a expansion/contraction factor acting on the entire structure. The same approach has recently successfully been used to model gold nanoclusters.^73^

#### *In situ* X-ray total scattering and data treatment

An *in situ* X-ray total scattering experiment was performed using a modified version of the setup described in detail by Becker *et al.* ^74^ The reaction vessel in this case was a fused silica capillary (inner diameter 0.70 mm, outer diameter 0.85 mm). In contrast to the flow syntheses, a metal ion concentration of [Zn^2+^] = [W^6+^] = 0.50 M was used as a means to achieve better signal to noise ratio with the time resolution of 1 s and a *Q*_max_ of 17 Å^–1^ due to the short exposure time. The hydrothermal synthesis was performed at 300 °C and a solvent pressure of 250 bar. The *in situ* experiment was conducted at beamline P02.1, PETRA III, DESY, Hamburg, Germany, using the same experimental geometry, wavelength, and sample-to-detector distance as the aforementioned *ex situ* experiment.

### UV-VIS DRS

Optical diffuse reflectance measurements (DRS) were performed at room temperature using a Shimadzu UV-3101 PC spectrometer operating in the 200–700 nm range (details of data treatment are given in the ESI†).

### TEM/HR-TEM/STEM-EDS

Transmission electron microscopy (TEM) images were recorded on a Philips CM20 electron microscope equipped with a LaB_6_ filament at 200 kV while HR-TEM, STEM and STEM-EDS data were obtained on a TALOS F200A. Further experimental details are given in the ESI.†
