# Supplementary material for: The chemistry of ZnWO4 nanoparticle formation
Source: Chem Sci. 2016 Jul 5;7(10):6394–406. doi: 10.1039/c6sc01580h (PMC5355961; doi:10.1039/c6sc01580h)
Supplement: Supplementary file 1 [file SC-007-C6SC01580H-s001.pdf]

Electronic Supplementary Information for

The chemistry of $ZnWO_4$ nanoparticle formation

Espen D. Bøjesen,[a] Kirsten M.Ø. Jensen,[b] Christoffer Tyrsted,[c] Aref Mamakhel,[a] Henrik L. Andersen,[a] Hazel Reardon,[a] Jacques Chevalier,[d] Ann-Christin Dippel[e] and Bo B. Iversen[a]*

[a]Center for Materials Crystallography, Department of Chemistry and iNANO, Aarhus University, Langelandsgade 140, DK-8000, Aarhus, Denmark

[b]Department of Chemistry, University of Copenhagen, 2100 København Ø, Denmark

[c]Haldor Topsøe A/S, Haldor Topsøes Allé 1, 2800 Kgs. Lyngby, Denmark

[d]Department of Physics and Astronomy, Aarhus University, Ny Munkegade 120, DK-8000 Aarhus C, Denmark

[e]Deutsches Elektronen-Synchrotron DESY, Photon Science Division, Notkestrasse 85, D-22607 Hamburg, Germany

Experimental details

Flow synthesis

The reactant solution (precursor-water slurry) was pumped at a rate of 10 ml/min using an injector device in combination with a solvent pump pumping deionized water at a rate of 15 ml/min through a heated pre-reactor zone. $ZnWO_4$ nanoparticles were synthesized at an internal reactor temperature and solvent temperature of 250, 300, 350 and 400 ˚C, respectively. The internal pressure was fixed at 250±10 bar for all syntheses. The as-prepared nanomaterial suspensions had a pH of ~7. The final product was centrifuged and washed with deionized water three times, and subsequently dried under vacuum at 40 ºC for 24 hours. In addition, a precursor powder sample was prepared from the white precursor gel by the same centrifuge–washing and drying process as the nanocrystalline product.

PXRD measurements

The beamline used a large image plate detector in Debye Scherrer geometry and a wavelength of 0.499818 (4) Å. The powders were filled in glass capillaries with a diameter of 0.3 mm, ensuring high intensities while minimizing absorption and capillary diameter related contributions to the instrumental profile. The capillary was rotated during measurements to improve powder statistics. The samples were cooled to 100 K by a N_2 cryostream device.

Due to the short wavelength, thin capillary diameter, and the nanosized nature of the crystallites, no absorption correction was applied. The anisotropic size broadening of the diffraction peaks was described using an empirical approach based on the Scherrer formula and a linear combination of spherical harmonics previously used for similar cases (the final shapes obtained from refinements are shown in the SI).[1-4] The profile function employed was the Thompson-Cox-Hastings Pseudo-Voigt function,[5] allowing for axial divergence asymmetry. Additionally, to obtain proper fits of the peak profiles, an isotropic microstrain contribution was modeled by refining one Gaussian (U) and one Lorentzian (X) term, both having a $\tan^2(\theta)$ dependency. Using this model the maximum apparent microstrain, as defined by Stokes and Wilson, could be determined.[6] The instrumental contribution to the peak profiles was determined by Rietveld refinements of NIST standard CeO_2 sample to determine the instrumental resolution function. A range of refinement strategies were tried in an effort to obtain the most reliable fit whilst optimizing the amount of information extracted for all samples. Table S1 summarizes the refinement results of the chosen model; similar tables for two other tested models are listed in Table S2 and Table S3. The atomic displacement parameters (ADPs) of oxygen were fixed at the 100 K values (measurement T) reported by Trots *et al.*[7], while the ADPs of Zn and W were refined isotropically.

An anti-site defect was incorporated in the model to properly account for the intensities of the observed reflections. The defect was implemented by refining the compositions of the 2*f* site (Zn in the bulk structure) and 2*e* site (W in the bulk structure), respectively. To ensure charge balance and simultaneously retain stoichiometry, full occupancy on both sites was assumed and the refinements were carried out with these constraints in place. An antisite concentration of, e.g. 3 % thus means that the composition of the 2*f* site is 97% Zn and 3% W and accordingly the 2*e* site has a composition of 97% W and 3% Zn.

The measurements were performed on the exact same flow-synthesized samples as the high resolution synchrotron PXRD investigations. Glass capillaries were used as sample containers, and total scattering data of the empty capillaries were collected for background subtraction during data analysis. A wavelength of 0.18896 Å and a sample to detector distance of 102.84 mm were used. The 14-bit dynamic Frelon4M CCD detector (50x50 µm pixel size) was placed off-centre, which provided a useful Q_{max} of 21 $Å^{-1}$. The experimental Q_{damp} was determined to be 0.0215 $Å^{-1}$ by refinement in PDFgui[8] of data from a

LaB_6-calibrant measurement. Exposure times were chosen individually for each sample ensuring the highest signal-to-noise ratio within the dynamic range of the detector and were on the order of 10-30 seconds.

X-ray total scattering measurements on the dried and washed precursor were performed at beamline P02.1, PETRA III, DESY, Hamburg, Germany.[9] A Perkin Elmer XRD 1621 amorphous silicon detector placed at a sample-to-detector distance of 188.1 mm was used. An X-ray wavelength of 0.20727 Å was applied for all measurements. This setup produced a usable Q_{max} of 18 $Å^{-1}$. The experimental Q_{damp} was found to be 0.02246 $Å^{-1}$, and the exposure time was approximately 2 min. It has to be noted that *ex situ* total scattering data were collected at 300 K and not at 100 K as was the case for the PXRD data.

UV-VIS-DRS

Diffuse reflectance spectra were used to estimate the band gap of the materials by converting reflectance curves to absorption curves according to the Kubelka-Munk function, assuming direct allowed transitions: $\alpha/S = (1-R)^2(2R)^{-1}$, where R is the reflectance and α and S are the absorption and scattering coefficients, respectively.[10-12]

Electron microscopy and EDS

Particle sizes obtained from TEM images were estimated using the software FIJI (v.1.49q).[13] HR-TEM images were obtained on a TALOS F200A with a TWIN lens system, X-FEG electron source, Ceta 16M Camera and a Super-X EDS Detector. Spatially resolved elemental analysis, with a spatial resolution better than 2 nm, was obtained using the same TALOS microscope in STEM mode. Exposure times of 5 minutes were used to create elemental distribution maps with satisfactory counting statistics, while minimizing potential problems such as beam damage and specimen drift. STEM pictures were obtained using a High Angle Annular Dark Field detector (HAADF). RGB overlays of the STEM EDX elemental maps were made using the FIJI (v.1.49q) software. [13]

Table S1. This table contains the refined values for the model chosen. In this model the following parameters were refined:

Unit cell parameters, scale factor, zero point displacement, atomic positions, profile parameters for size and microstrain, isotropic atomic displacement parameters for W and Zn. Finally, the compositions of the 2*f* and 2*e* sites were refined under the constraint of full total occupancy of both sites, for details see description earlier in the SI.

Synthesis T		250 ºC	300 ºC	350 ºC	400 ºC	Bulk
a (Å)		4.67656 (9)	4.67585 (6)	4.67755 (4)	4.67919 (2)	4.6839
b (Å)		5.73335 (9)	5.72473 (7)	5.71499 (4)	5.70959 (2)	5.7101
c (Å)		4.94730 (9)	4.93677 (7)	4.92513 (4)	4.92423 (2)	4.9223
β (º)		9.493 (1)	90.526 (1)	90.550 (1)	90.554 (1)	90.558
Zn (2f)	*y/b*	0.6775 (3)	0.6802 (2)	0.6830 (1)	0.6832 (1)	0.6828 (3)
W (2e)	*y/b*	0.1775 (1)	0.17939 (8)	0.18118 (6)	0.18166 (4)	0.1810 (4)
O1 (4g)	*x/a*	0.2303 (9)	0.2255 (7)	0.2216 (6)	0.2171 (4)	0.2165 (2)
	y/b	0.8880 (9)	0.8914 (7)	0.8925 (5)	0.8934 (4)	0.8954 (2)
	z/c	0.416 (1)	0.4270 (9)	0.4388 (8)	0.4384 (5)	0.4367 (2)
O2 (4g)	*x/a*	0.2558 (9)	0.2577 (7)	0.2578 (6)	0.2550 (4)	0.2564 (2)
	y/b	0.370 (1)	0.3714 (8)	0.3729 (6)	0.3764 (4)	0.3756 (2)
	z/c	0.392 (1)	0.3956 (9)	0.3984 (7)	0.3993 (5)	0.3997 (3)
Occ (Zn @ Zn)		0.966 (1)	0.972 (1)	0.976 (1)	0.982 (1)	1
Occ (W @ W)		0.966 (1)	0.972 (1)	0.976 (1)	0.982 (1)	1
Occ (W @ Zn)		0.034 (1)	0.028 (1)	0.024 (1)	0.018 (1)	0
Occ (Zn @ Zn)		0.034 (1)	0.028 (1)	0.024 (1)	0.018 (1)	0
Biso (Zn)		0.45 (3)	0.41 (2)	0.30 (1)	0.214 (9)	0.12 (3)
Biso (W)		0.55 (1)	0.41 (1)	0.215 (6)	0.174 (4)	0.31 (4)
Size (*a*)		20.6 (2)	24.1 (3)	27.7 (3)	29.1 (1)	N/A
Size (*b*)		12.2 (3)	14.7 (2)	17.9 (2)	23.7 (2)	N/A
Size (c)		16.3 (3)	21.8 (3)	32.2 (4)	42.3 (3)	N/A
Microstrain		21.0 (2)	22.2 (3)	15.7 (3)	6.7 (2)	N/A
R_B (%)		1.99	1.52	1.22	1.11	N/A
R_F (%)		1.36	1.01	0.69	0.55	N/A
R_p (%)		2.31	4.20	3.75	2.95	4.81
R_{wp} (%)		3.31	5.03	4.47	3.60	5.86
χ^2		4.98	4.55	4.65	3.11	2.54

Table S2. This table contains the refined values for another tested model. In this model the following parameters were refined:

Unit cell parameters, scale factor, zero point displacement, atomic positions, profile parameters for size and microstrain, isotropic atomic displacement parameters for W, Zn, O1 and O2. Moreover, all occupancies except for the O1 site were refined without constraints. For the samples synthesized at low T this approach led to unphysical ADPs for Oxygen as well as unphysically high occupancies for various sites, thus the model was not used.

Synthesis T		250 ˚C	300 ˚C	350 ˚C	400 ˚C	Bulk
a (Å)		4.67641 (9)	4.67582 (6)	4.67751 (4)	4.67917 (2)	4.6839(1)
b (Å)		5.73327 (9)	5.72471 (7)	5.71486 (4)	5.70952 (2)	5.7101(1)
c (Å)		4.94718 (9)	4.93673 (7)	4.92502 (4)	4.92419 (2)	4.9223(1)
β (o)		90.490 (1)	90.525 (1)	9.550 (1)	90.554 (1)	90.558(1)
Zn (2f)	y/b	0.6786 (3)	0.6803 (2)	0.6819 (2)	0.6823 (1)	0.6828 (3)
W (2e)	y/b	0.1774 (1)	0.17951 (9)	0.18161 (6)	0.18199 (4)	0.1810 (4)
O1 (4g)	x/a	0.2297 (9)	0.2245 (7)	0.2212 (5)	0.2167 (4)	0.2165 (2)
	y/b	0.8895 (9)	0.8913 (7)	0.8910 (5)	0.8920 (3)	0.8954 (2)
	z/c	0.412 (2)	0.425 (1)	0.4397 (7)	0.4391 (4)	0.4367 (2)
O2 (4g)	x/a	0.2558 (9)	0.2245 (7)	0.2569 (6)	0.2538 (4)	0.2564 (2)
	y/b	0.3689 (9)	0.3714 (8)	0.3726 (6)	0.3771 (4)	0.3756 (2)
	z/c	0.392 (1)	0.3938 (9)	0.3962 (7)	0.3977 (4)	0.3997 (3)
Occ (Zn)		1.12 (7)	1.14 (1)	0.922 (5)	0.912 (4)	1
Occ (W)		1.04 (7)	0.95 (1)	0.866 (5)	0.866 (2)	1
Occ (O2)		1.09 (1)	1.02 (1)	0.931 (6)	0.934 (4)	1
Biso (Zn)		0.36 (2)	0.36 (2)	0.29 (1)	0.214 (8)	0.12 (3)
Biso (W)		0.59 (2)	0.41 (1)	0.204 (6)	0.164 (4)	0.31 (4)
Biso (O1)		-0.18 (1)	0.3 (1)	0.43 (8)	0.41 (5)	0.19 (2)
Biso (O2)		-0.582 (9)	-0.2 (1)	0.22 (8)	0.24 (5)	0.16 (2)
Size (a)		20.6 (3)	24.4 (3)	28.1 (4)	29.5 (5)	N/A
Size (b)		12.4 (3)	14.9 (2)	18.1 (3)	23.9 (4)	N/A
Size (c)		16.4 (2)	21.9 (3)	32.5 (3)	42.3 (3)	N/A
Microstrain		21.0 (4)	22.3 (4)	15.8 (3)	6.2 (2)	N/A
RB (%)		1.97	1.53	1.14	0.94	N/A
RF (%)		1.37	1.03	0.66	0.52	N/A
Rp (%)		4.86	4.17	3.67	2.79	4.81
Rwp (%)		5.94	4.99	4.36	3.40	5.86
$\chi 2$		4.89	4.48	4.43	2.86	2.54

Table S3. This table contains the refined values for the model chosen. In this model the following parameters were refined:

Unit cell parameters, scale factor, zero point displacement, atomic positions, and profile parameters for size and microstrain. Finally, the compositions of the 2*f* and 2*e* sites were refined under the constraint of full total occupancy of both sites, for details see description earlier in the SI. No ADPs were refined and the agreements were not as good as the ones reported for the chosen model (see table S1)

Synthesis T		250 ˚C	300 ˚C	350 ˚C	400 ˚C	Bulk
a (Å)		4.67610 (9)	4.67563 (6)	4.67756 (4)	4.67922 (2)	4.6839(1)
b (Å)		5.73290 (9)	5.72457 (7)	5.71500 (4)	5.70959 (3)	5.7101(1)
c (Å)		4.94727 (9)	4.93674 (7)	4.92513 (4)	4.92424 (2)	4.9223(1)
β (o)		90.487 (1)	90.524 (1)	90.551 (1)	90.554 (1)	90.558(1)
Zn (2f)	y/b	0.6776 (3)	0.6803 (2)	0.6831 (2)	0.6832 (1)	0.6828 (3)
W (2e)	y/b	0.1777 (1)	0.17944 (8)	0.18115 (6)	0.18263 (4)	0.1810 (4)
O1 (4g)	x/a	0.2315 (9)	0.2272 (7)	0.2232 (6)	0.2181 (4)	0.2165 (2)
	y/b	0.8875 (9)	0.8907 (7)	0.8922 (5)	0.8934 (4)	0.8954 (2)
	z/c	0.419 (2)	0.428 (1)	0.4384 (7)	0.4378 (5)	0.4367 (2)
O2 (4g)	x/a	0.2580 (9)	0.2595 (7)	0.2588 (6)	0.2559 (4)	0.2564 (2)
	y/b	0.374 (1)	0.3725 (8)	0.3733 (6)	0.3763 (4)	0.3756 (2)
	z/c	0.390 (1)	0.396 (1)	0.3992 (7)	0.4002 (5)	0.3997 (3)
Occ (Zn @ Zn)		0.970 (1)	0.978 (1)	0.984 (1)	0.988 (1)	1
Occ (W @ W)		0.970 (1)	0.978 (1)	0.984 (1)	0.988 (1)	1
Occ (W @ Zn)		0.030 (1)	0.022 (1)	0.016 (1)	0.012 (1)	0
Occ (Zn @ Zn)		0.030 (1)	0.022 (1)	0.016 (1)	0.012 (1)	0
Size (a)		22.9 (3)	25.5 (2)	26.9 (2)	27.7 (4)	N/A
Size (b)		13.7 (2)	15.6 (2)	17.4 (3)	22.4 (3)	N/A
Size (c)		19.2 (3)	23.9 (3)	31.2 (3)	39.4 (5)	N/A
Microstrain		24.7 (3)	24.4 (4)	15.3 (3)	7.0 (2)	N/A
RB (%)		1.74	1.44	1.37	1.42	N/A
RF (%)		0.85	0.84	0.99	1.21	N/A
Rp (%)		4.59	4.10	3.97	3.38	4.81
Rwp (%)		5.90	5.02	4.63	3.93	5.86
$\chi 2$		5.35	4.77	4.82	3.49	2.54

Table S4. This table contains the refined values for the model chosen. In this model the following parameters were refined:

Unit cell parameters, scale factor, zero point displacement, atomic positions, and profile parameters for size and microstrain. This approach led to poorer agreement factors than the chosen model (Table S1) and was thus not chosen.

Synthesis T		250 °C	300 °C	350 °C	400 °C	Bulk
a (Å)		4.6756 (1)	4.67544 (7)	4.67748 (4)	4.67920 (2)	4.6839
b (Å)		5.7328 (1)	5.72454 (7)	5.71499 (5)	5.70957 (3)	5.7101
c (Å)		4.9473 (1)	4.93680 (7)	4.92516 (5)	4.92424 (2)	4.9223
β (°)		90.491 (1)	90.525 (1)	90.551 (1)	90.554 (1)	90.558
Zn (2f)	*y/b*	0.6775 (4)	0.6801 (2)	0.6828 (2)	0.6831 (1)	0.6828 (3)
W (2e)	*y/b*	0.1777 (1)	0.17956 (9)	0.18126 (5)	0.18167 (5)	0.1810 (4)
O1 (4g)	*x/a*	0.2425 (9)	0.2333 (7)	0.2246 (5)	0.2201 (5)	0.2165 (2)
	y/b	0.8812 (9)	0.8878 (8)	0.8912 (6)	0.8928 (4)	0.8954 (2)
	z/c	0.408 (2)	0.425 (1)	0.4376 (8)	0.4371 (5)	0.4367 (2)
O2 (4g)	*x/a*	0.264 (1)	0.2633 (8)	0.2614 (6)	0.2578 (4)	0.2564 (2)
	y/b	0.377 (1)	0.3755 (9)	0.3745 (6)	0.3771 (5)	0.3756 (2)
	z/c	0.390 (2)	0.398 (1)	0.4015 (8)	0.4015 (5)	0.3997 (3)
Size (*a*)		21.9 (3)	24.7 (3)	26.3 (3)	27.3 (3)	N/A
Size (*b*)		13.2 (2)	15.3 (2)	17.1 (3)	22.2 (3)	N/A
Size (c)		19.0 (3)	24.1 (3)	31.2 (4)	49.2 (4)	N/A
Microstrain		24.5 (2)	24.3 (2)	15.5 (2)	7.1 (2)	N/A
R_B (%)		1.88	1.63	1.56	1.56	N/A
R_F (%)		0.89	0.86	0.98	1.20	N/A
R_p (%)		4.78	4.31	4.19	3.51	4.81
R_{wp} (%)		6.24	5.31	4.86	4.10	5.86
χ^2		6.09	5.39	5.32	3.90	2.54

Fig S0: The refined isotropic B values for Zn and W from the chosen model (Table S1) plotted as a function of synthesis themperature. The two values shown at 405 degrees are the reported bulk values. The trend of decreasing B values as a function of synthesis temperature is in accordance with the observed trend in decreasing defect and disorder as function of synthesis temperature. Thus the large B values for the low temperature samples are more likely a manifestation of modelling static disorder than dynamic disorder.

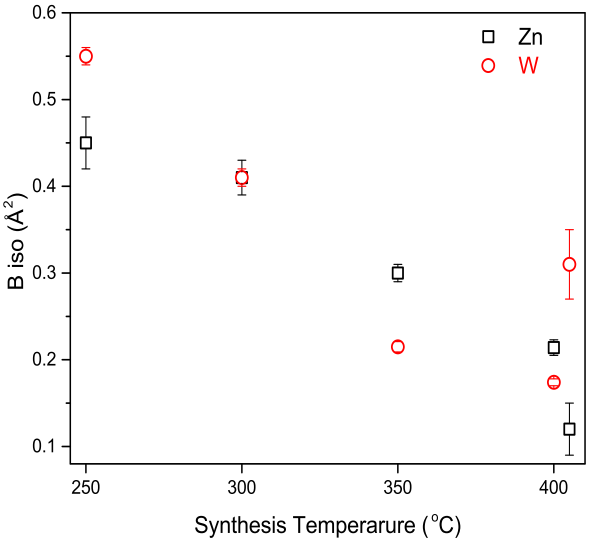

Real space refinements of the dried $ZnWO_4$ samples prepared in the program PDFgui. For all samples the occupancies were kept fixed at the standard values and the parameters were constrained to follow the symmetry of P 2/c.

Fig S1

A) Fit of dry 250 ˚C sample

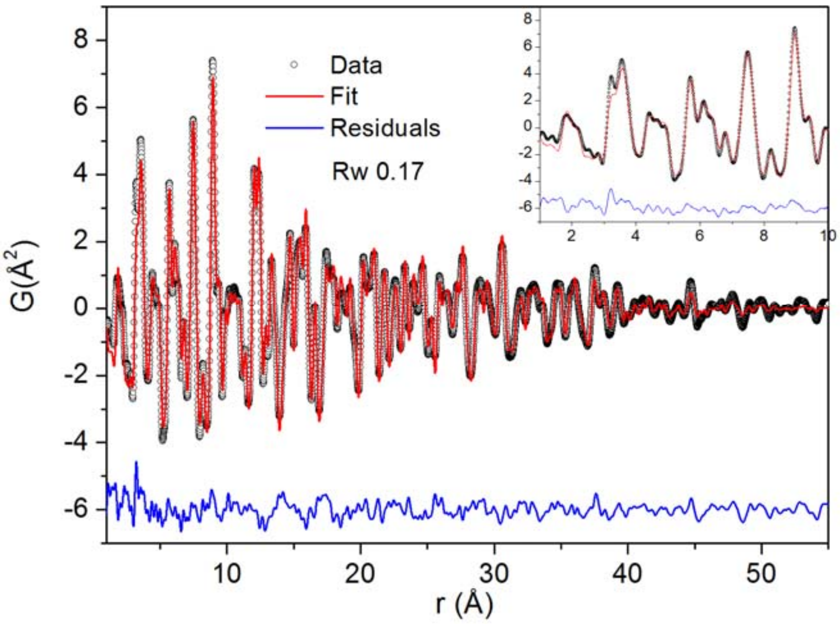

atom	x	y	z	U11	U22	U33	U12	U13	U23
Zn1	0.50000	0.32189	0.75000	0.01144	0.01757	0.01285	0.00000	0.00084	0.00000
W1	0.00000	0.81924	0.75000	0.00694	0.00730	0.00537	0.00000	0.00102	0.00000
O1	0.22411	0.11093	0.93474	0.08678	0.00400	0.01146	-0.00158	0.01572	-0.00326
O2	0.26326	0.62813	0.88974	0.02376	0.02068	0.02843	-0.01011	0.00555	-0.00102

Unit cell parameters: *a*= 4.6728 Å; *b*= 5.7249 Å; *c*= 4.9315 Å, β=90.62˚

B) Fit of dry 300 ˚C sample

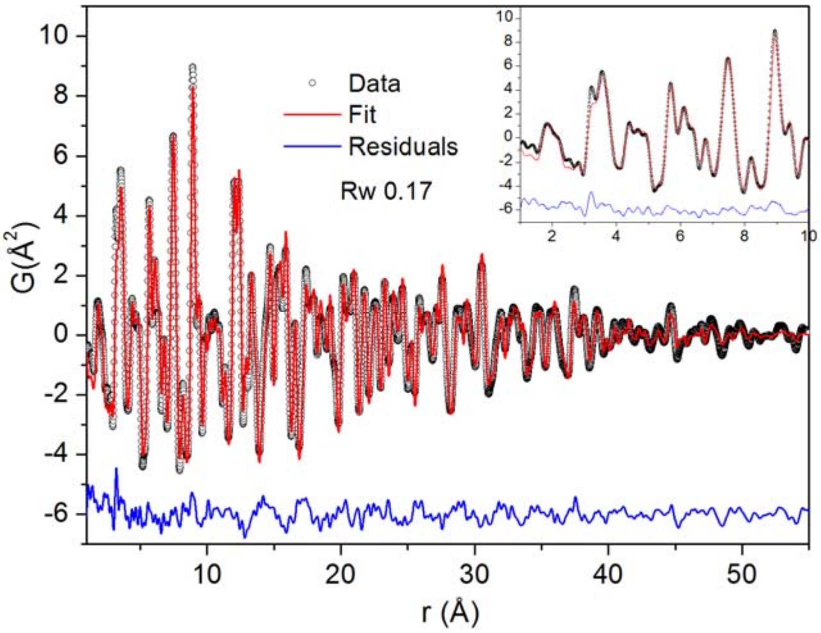

atom	x	y	z	U11	U22	U33	U12	U13	U23
Zn1	0.50000	0.32014	0.75000	0.00998	0.01552	0.01060	0.00000	0.00108	0.00000
W1	0.00000	0.81846	0.75000	0.00582	0.00621	0.00484	0.00000	0.00069	0.00000
O1	0.21847	0.11047	0.93259	0.08091	0.00626	0.00961	-0.00488	0.01152	-0.00477
O2	0.26003	0.63070	0.89498	0.02434	0.01844	0.02887	-0.00510	0.00845	-0.00088

Unit cell parameters: *a*= 4.6722 Å; *b*= 5.7163 Å; *c*= 4.9202 Å, β=90.60˚

C) Fit of dry 350 ˚C sample (Rw 0.17)

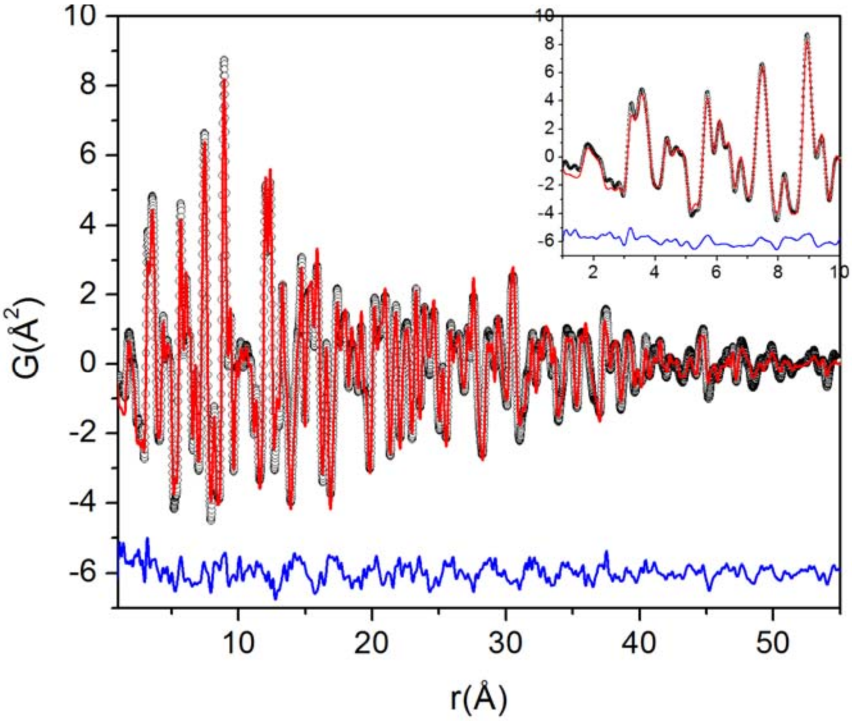

atom	x	y	z	U11	U22	U33	U12	U13	U23
Zn1	0.50000	0.31797	0.75000	0.00929	0.01105	0.01211	0.00000	0.00163	0.00000
W1	0.00000	0.81804	0.75000	0.00537	0.00514	0.00409	0.00000	0.00034	0.00000
O1	0.21309	0.10874	0.10874	0.08262	0.00556	0.01192	-0.00305	0.01344	-0.00375
O2	0.25798	0.63027	0.88632	0.02437	0.01724	0.02143	-0.00131	0.00658	-0.00438

Unit cell parameters: ***a*= 4.6783 Å; *b*= 5.7165 Å; *c*= 4.9176 Å, β=90.61˚**

D) Fit of 400 ˚C dry sample

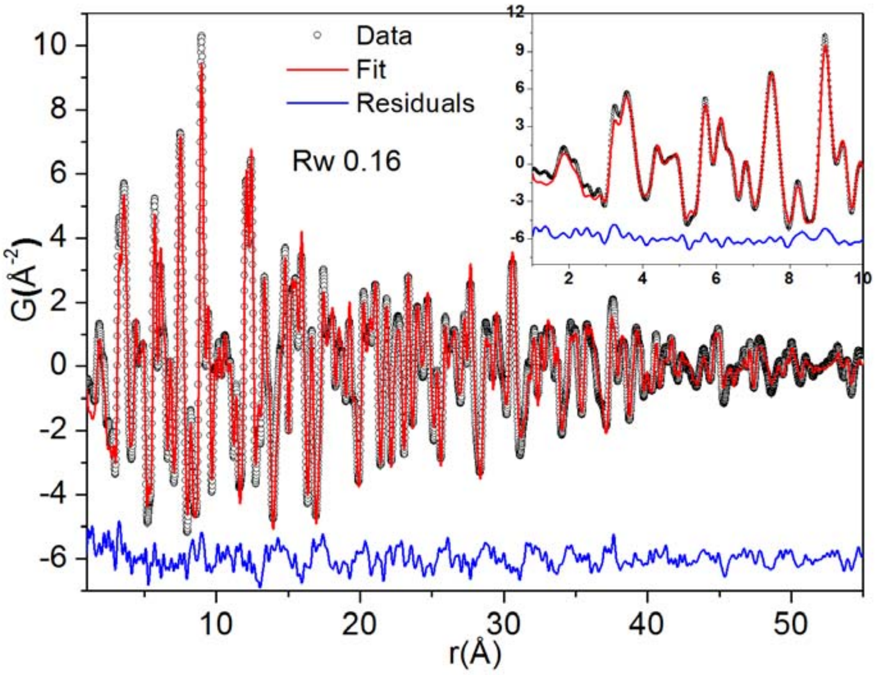

atom	x	y	z	U11	U22	U33	U12	U13	U23
Zn1	0.50000	0.31647	0.75000	0.00915	0.01079	0.00830	0.00000	0.00109	0.00000
W1	0.00000	0.81767	0.75000	0.00553	0.00544	0.00328	0.00000	0.00019	0.00000
O1	0.19885	0.11228	0.92826	0.12863	0.00505	0.01251	-0.01373	0.02971	-0.00532
O2	0.25831	0.63353	0.88720	0.02730	0.02238	0.03583	-0.00815	0.01388	-0.01228

Unit cell parameters: *a*=4.6930 Å; *b*=5.7289 Å; *c*= 4.9356 Å, β=90.59˚

Fig S2. Example of the refined crystallite shapes based on Rietveld refinements

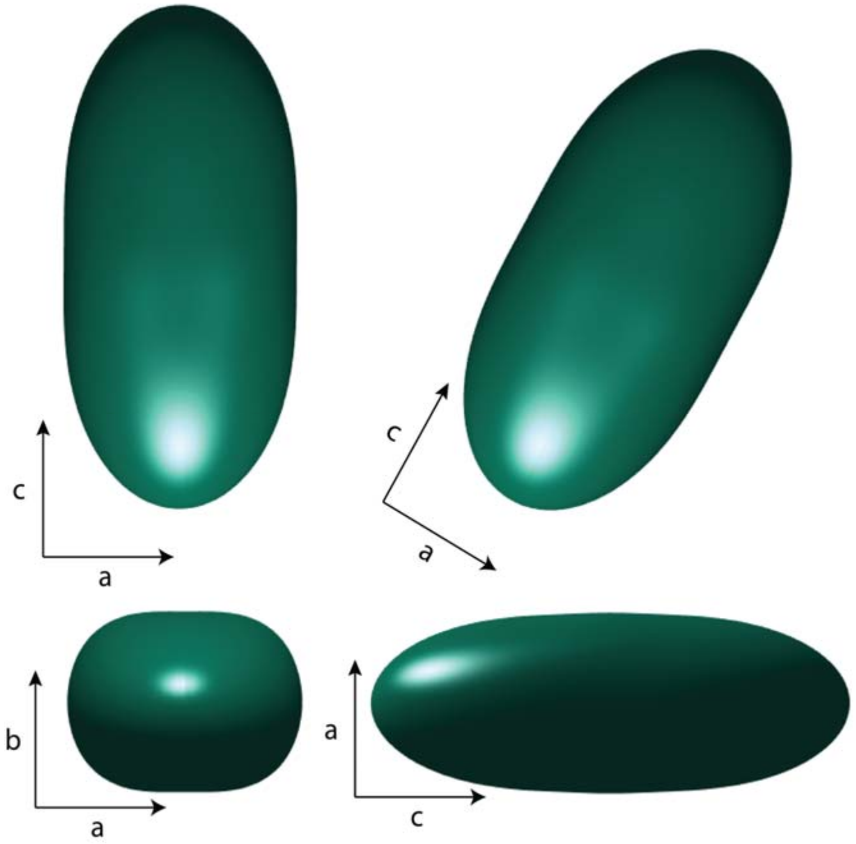

The size contribution to the integral breadth of the PXRD profiles was modelled using the following implementation of the normalized (as described in Jarvinen, J. App. Cryst, (1993), 26, 525-531) spherical harmonic functions in Fullprof.

$$\beta_{hkl} = \frac{\lambda}{D_{hkl}\cos\theta} = \sum_{lmp} a_{lmp} y_{lmp}(\boldsymbol{\Theta}_{hkl}\,, \boldsymbol{\Phi}_{hkl})$$

The arguments to the functions are the polar angles of the vector [hkl] with respect to the spherical coordinate system, as described in Jarvinen, J. App. Cryst, (1993), 26, 525-531. Θ is the inclination angle (defined to go from the z-axis to the directional vector to the the xy-plane), while Φ is the azimuthal angle (from the xz-plane to the vector towards the y-axis). The sizes along different crystallographic directions (D_{hkl}) thus can be extracted from this equation by refining the coefficients a_{lmp} of each selected spherical harmonic function (y_{lmp}), taking into account the instrumental resolution and any microstrain contribution. The spherical harmonics used are based on the symmetry of the Laue group of the parent crystal structure. The crystallite shapes shown in Fig S2 have been plotted using MATlab.

Fig S3 PDFs of both the dry and "wet", i.e., freshly mixed, precursor. Moreover an overlay of the two as well as an overlay with the structure after 220s of heating is shown. Minor differences between the wet and dry precursor structure exist, in particular it can be seen that a difference in the ratio of the M-M double peak exist. In both definite, albeit broad, peaks are observed out to at least 10-11 Å. This is thus longer distances than what is expected from isolated Keggin clusters.

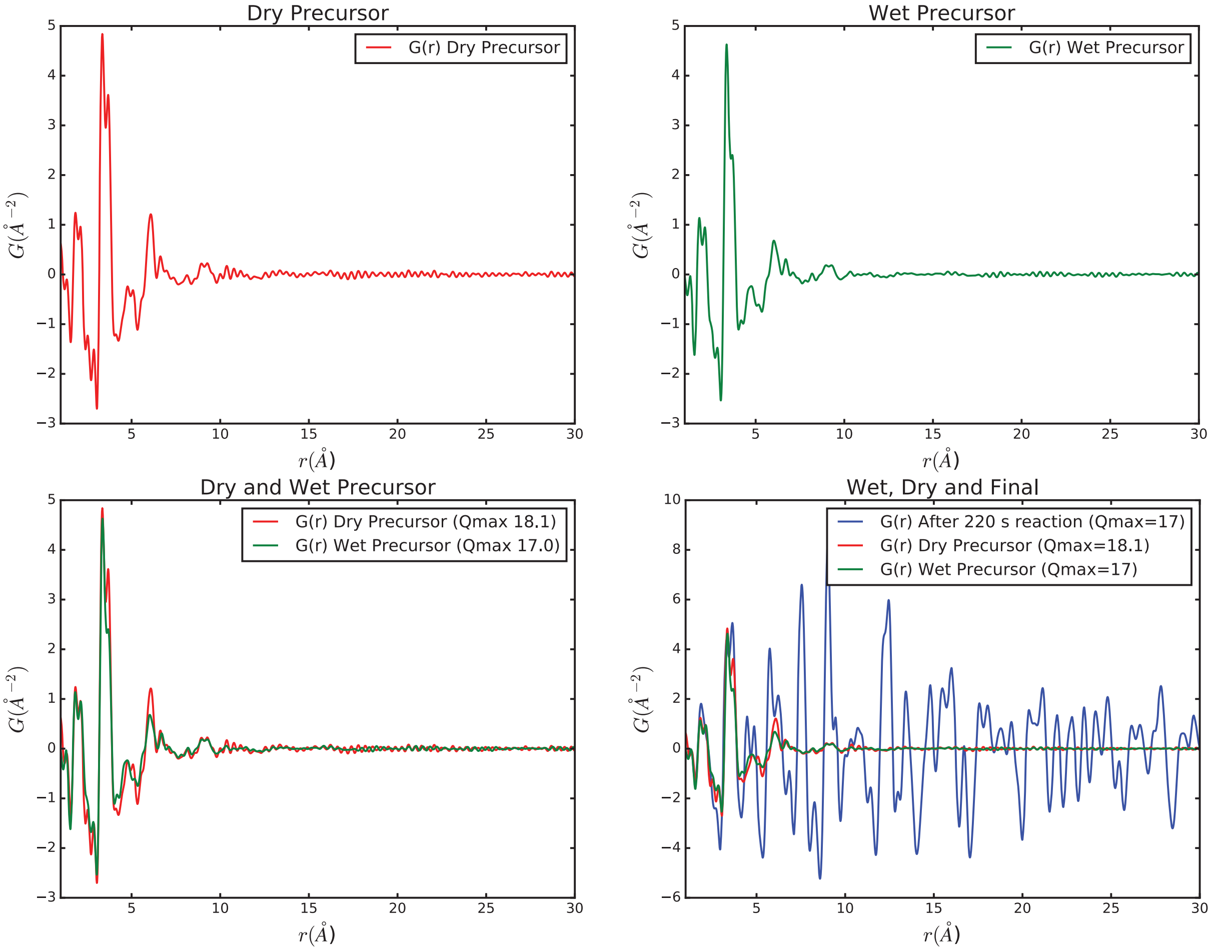

Fig S4 Fit of the wet precursor using the same model as for the dry precursor. The fit is very (Rw=0.24) good and the same atomic coordinates as for the dry precursor refinement (see Table S7) are used. The only difference here are the B iso values (some extremely large) as well as the values for correlated motion (which here is unphysically large. These are as follows:

Delta2 = 10.2 , Biso Zn = 0.4 , Biso W= 6.2, Biso O = 0.6, Biso Na = 0.09

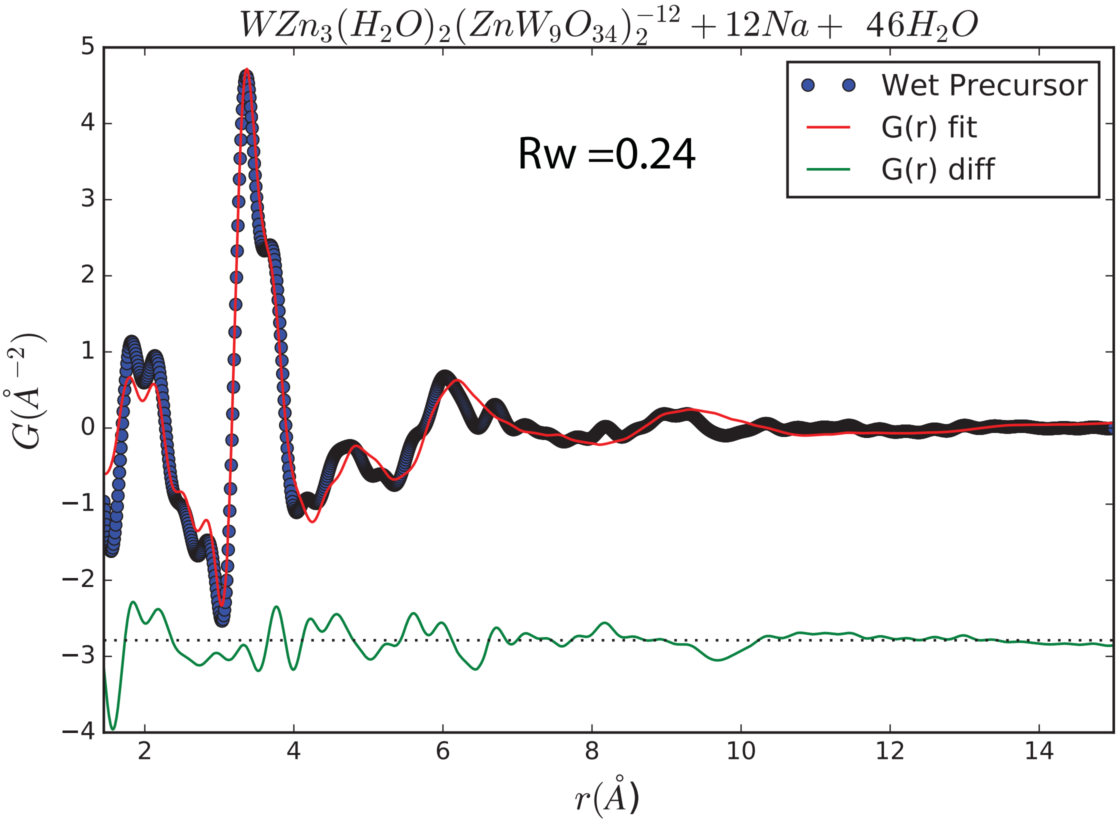

Thus the wet precursor definitely shows similar features as the dry one, albeit with an even larger degree of disorder. The process of drying may induce some ordering in the structure due to loss of disordered water molecules.

Fig S5 Example of the double Gauss-fit of the M-M correlation peak for the data obtained after four seconds of reaction. The blue crosses are the baseline corrected (linear baseline) data, the red line is the total fit, the black lines show the individual Gauss curves of the fit, the red and orange dots indicate the areas used for calculating the integrated area under each curve.

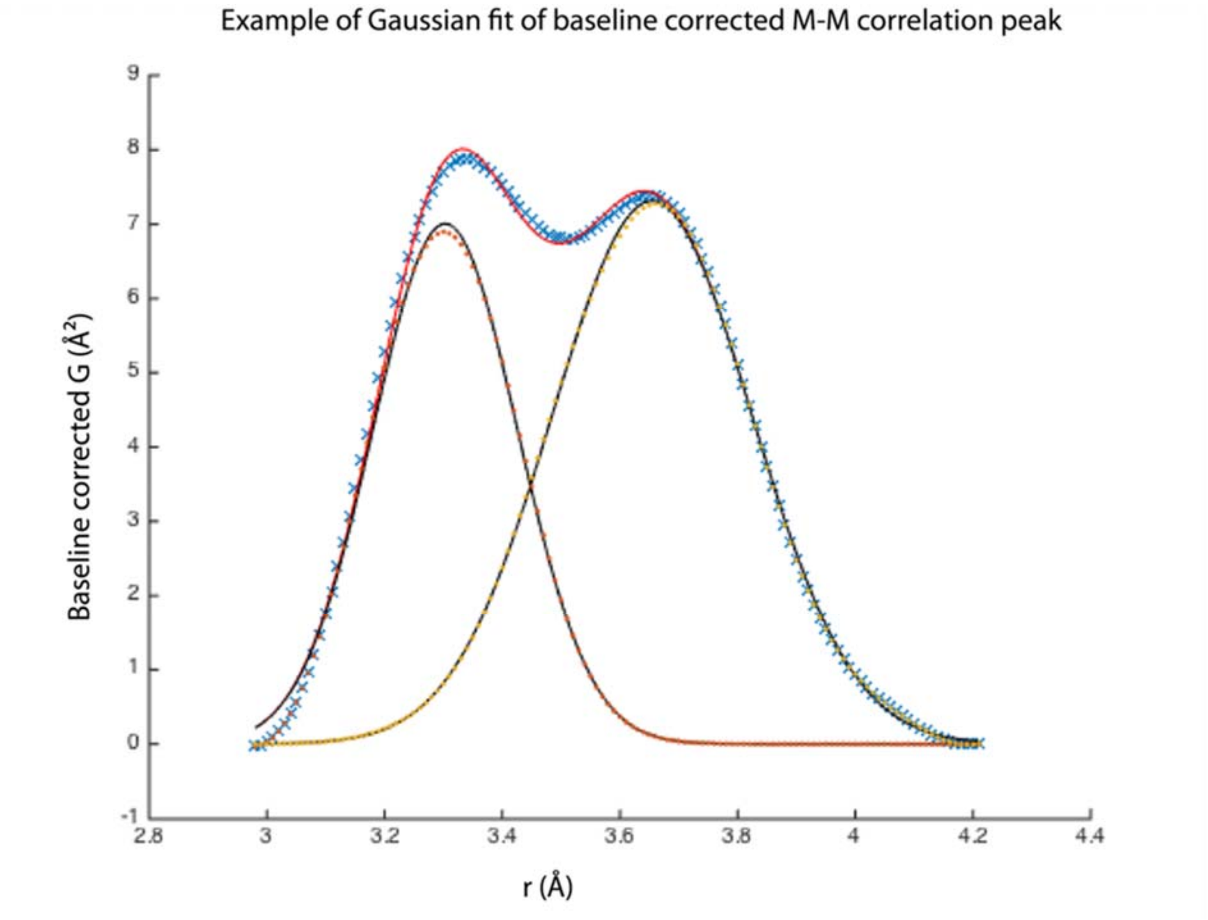

General model Gauss2:

fitresult(x) = a1*exp(-((x-b1)/c1)^2) + a2*exp(-((x-b2)/c2)^2)

Coefficients (with 95% confidence bounds):

a1 =7.012 (6.89, 7.133), b1 = 3.303 (3.3, 3.306), c1 = 0.1731 (0.17, 0.1763)

a2 =7.315 (7.253, 7.376), b2 = 3.655 (3.65, 3.659)

c2 = 0.2397 (0.2351, 0.2443)

integrated_intensity = 2.1233 and 3.1039 ratio = 0.6840 $y_{obs,i} = \frac{y_i}{y_1+y_2} * y_{obs}$ $I_{obs,i} = \sum y_{obs,i}$

UV-VIS-NIR-DRS

The normalized Kubelka-Munk function curves obtained from UV-VIS-DRS measurements from the $ZnWO_4$ particles are shown in Figure 8, where the determined bandgaps (based on Tauc plots) are stated in the graph. The bandgap was found to decrease with increasing synthesis temperature, which is most likely related to the differences in bond length and antisite concentration observed. Furthermore, a number of features in the curves obtained may be attributed to presence of defects or dynamic disorder. Generally, two (or more) slopes are observed for all samples in the plots of the normalized Kubelka-Munk function and the features at high energies differ from sample to sample, indicating some disorder. Furthermore, the shape of the curve of the sample synthesized at 400 ˚C differs significantly from the other curves. This could be a manifestation of the small W-rich impurities observed in the samples, and may suggest that the higher temperature induces a change in the structure and size of the impurities, resulting in the distinct UV-VIS-DRS spectrum of the 400 °C sample. The dried precursor sample exhibited a significantly wider band gap than the crystalline products and the shape of the curve for the precursor exhibits the typical trademarks observed for amorphous samples.

The previously reported dependence of photocatalytic activity of the compound on crystallite size and morphology may, accordingly, be understood on the basis of changes in the local M-O environment related to differences in nanostructure and defects and to different activities of exposed crystallite facets.

Fig S6

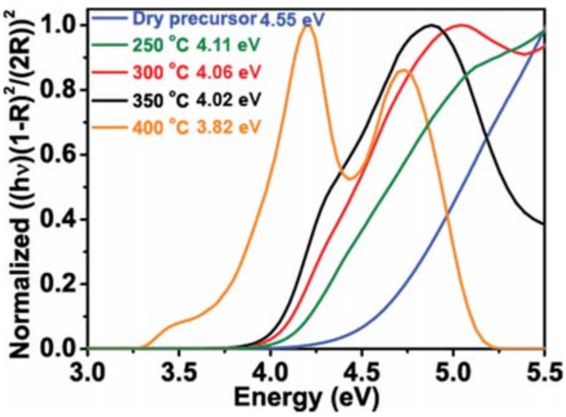

Plots of the normalized Kubelka-Munk function. The bandgaps stated in the legend are based on fits of the constant gradient sections of the curves; for curves with two sections of constant gradient the mean value is stated.

Enlarged views of the FFT shown in Figure 8

Fig S7

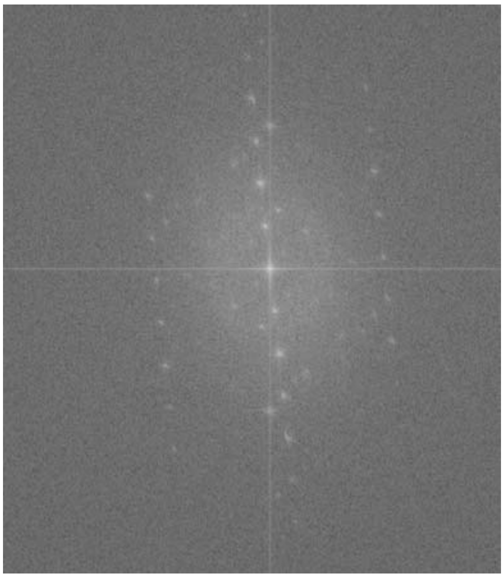

FFT shown in Figure 8 B

Fig S6

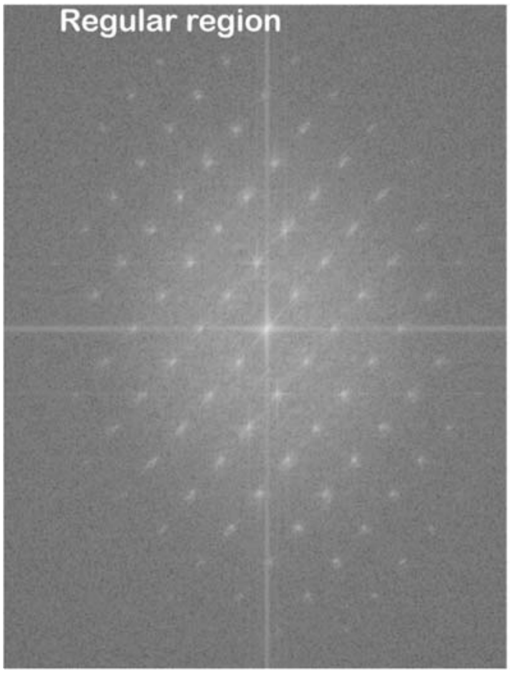

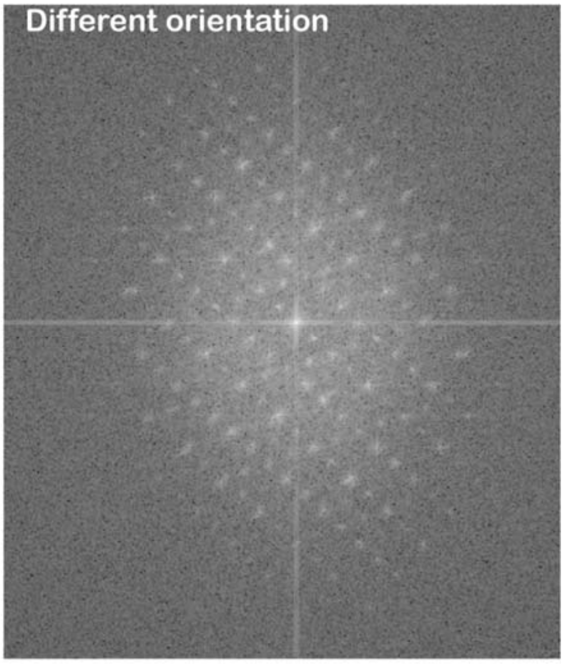

FFTs shown in Figure 8 D

Fit of the Precursor structure

In the precursor model all ADPs were refined isotropically and constrained together for atoms of the same type in similar position, e.g., W in the two halves of the sandwich. For the Na atoms only one common ADP was refined, the same is true for the O atoms. The positions of the Zn and W atoms were refined using the symmetry constraints of the spacegroup $P2_1/c$. The positions of the Na and O atoms were not refined. The "unit cell parameters" of the structure were also refined with the constraints applying to the spacegroup.

"unit cell parameters"

***a*=12.401 Å, *b*=17.551 Å, *c*=24.793 Å, α= 90˚, β=114.620 ˚, γ=90 ˚**

Spacegroup $P2_1/c$, SP-diameter 11.8 Å

Table S6

label	occ	x	y	z	U_{iso} (Å^2)
W1	0.6125	0.89395	0.05264	0.68021	1.85E-04
W2	0.6125	0.37799	0.21053	0.4703	1.85E-04
W3	0.6125	0.63927	0.0528	0.71529	1.85E-04
W4	0.6125	0.89792	0.23859	0.66945	1.85E-04
W5	0.6125	0.4054	0.12956	0.60599	1.85E-04
W6	0.6125	0.61956	0.24066	0.699	1.85E-04
W7	0.6125	0.61043	0.32341	0.55709	1.85E-04
W8	0.5	0.48691	0.046	0.42867	0.0047
W9	0.6125	0.63681	0.21955	0.44999	1.85E-04
W10	0.6125	0.89923	0.12595	0.56085	1.85E-04
Zn1	1	0.64815	0.13537	0.57814	0.00365
Zn2	0.3875	0.89395	0.05264	0.68021	1.85E-04
Zn3	0.3875	0.4054	0.12956	0.60599	1.85E-04
Zn4	0.3875	0.37799	0.21053	0.4703	1.85E-04
Zn5	0.5	0.48691	0.046	0.42867	0.00487
Zn6	1	0.2529	0.04591	0.46471	0.00365
Zn7	0.3875	0.63681	0.21955	0.44999	1.85E-04
Zn8	0.3875	0.89923	0.12595	0.56085	1.85E-04
Zn9	0.3875	0.63927	0.0528	0.71529	1.85E-04
Zn10	0.3875	0.61043	0.32341	0.55709	1.85E-04
Zn11	0.3875	0.61956	0.24066	0.699	1.85E-04
Zn12	0.3875	0.89792	0.23859	0.66945	1.85E-04
Na1	1	0.62908	0.42322	0.43633	2.37E-04
Na2	1	0.23072	0.381	0.6792	2.37E-04
Na3	1	0.6557	0.5601	0.6971	2.37E-04
Na4	1	0.75985	0.08544	0.28032	2.37E-04

Na5	1	0.3269	0.2483	0.2861	2.37E-04
Na6	1	0.07678	0.21349	0.41034	2.37E-04
O1	1	0.229	0.0541	0.6953	0.01689
O2	1	0.044	0.7692	0.3835	0.01689
O3	1	0.624	0.0662	0.3336	0.01689
O4	1	0.38	0.1969	0.5373	0.01689
O5	1	0.98	0.1318	0.3276	0.01689
O6	1	0.849	0.2028	0.3915	0.01689
O7	1	0.3	0.443	0.5057	0.01689
O8	1	0.424	0.438	0.4083	0.01689
O9	1	0.815	0.0516	0.5201	0.01689
O10	1	0.0259	0.0135	0.7606	0.01689
O11	1	0.437	0.239	0.4012	0.01689
O12	1	0.8061	0.1376	0.6166	0.01689
O13	1	0.763	0.4444	0.7109	0.01689
O14	1	0.7649	0.2973	0.604	0.01689
O15	1	0.982	0.3183	0.718	0.01689
O16	1	0.1	0.274	0.5081	0.01689
O17	1	0.616	0.4266	0.5592	0.01689
O18	1	0.3798	0.117	0.4477	0.01689
O19	1	0.213	0.1373	0.2723	0.01689
O20	1	0.141	0.1074	0.4672	0.01689
O21	1	0.434	0.1645	0.2549	0.01689
O22	1	0.585	0.301	0.6224	0.01689
O23	1	0.904	0.35	0.51	0.01689
O24	1	0.314	0.347	0.605	0.01689
O25	1	0.5976	0.0333	0.5334	0.01689
O26	1	0.4016	0.0453	0.5601	0.01689
O27	1	0.652	0.3105	0.7546	0.01689
O28	1	0.782	0.2409	0.7039	0.01689
O29	1	0.469	0.2196	0.6667	0.01689
O30	1	0.757	0.414	0.3995	0.01689
O31	1	0.929	0.0324	0.4274	0.01689
O32	1	0.238	0.2472	0.4128	0.01689
O33	1	0.489	0.479	0.6734	0.01689
O34	1	0.075	0.461	0.3813	0.01689
O35	1	0.961	0.324	0.3449	0.01689
O36	1	0.62	0.2476	0.3744	0.01689
O37	1	0.803	0.0676	0.7195	0.01689
O38	1	0.9747	0.161	0.7261	0.01689
O39	1	0.4858	0.0718	0.686	0.01689

O40	1	0.164	0.0194	0.367	0.01689
O41	1	0.684	0.0033	0.7792	0.01689
O42	1	0.27	0.1349	0.6033	0.01689
O43	1	0.3901	0.0182	0.3507	0.01689
O44	1	0.675	0.1571	0.7563	0.01689
O45	1	0.5709	0.2129	0.5185	0.01689
O46	1	0.5934	0.1395	0.6378	0.01689
O47	1	0.7698	0.1997	0.5007	0.01689
O48	1	0.6258	0.3259	0.4774	0.01689
O49	1	0.5789	0.1206	0.4298	0.01689
O50	1	0.463	0.3466	0.2893	0.01689
O51	1	0.992	0.1465	0.5323	0.01689
O52	1	0.9926	0.0814	0.6354	0.01689
O53	1	0.4408	0.3206	0.4972	0.01689
O54	1	0.215	0.3377	0.3043	0.01689
O56	1	0.203	0.2486	0.6722	0.01689

Table S7

Coordinates and isotropic B values for the Cluster used to fit the precursor structure using Diffpy CMI. Delta2 (correlated motion) = 6.3

#	label	x	y	z	Occ	A-Type	B iso
1	Na1	3.942	16.203	1.435	0.5	Na	4.5
2	Na2	8.458	1.348	-1.435	0.5	Na	4.5
3	Na3	6.174	6.687	-7.231	0.5	Na	4.5
4	Na4	6.226	10.864	7.231	0.5	Na	4.5
5	Na5	-1.01	15.462	-4.039	0.5	Na	4.5
6	Na6	11.39	15.462	-4.039	0.5	Na	4.5
7	Na7	1.01	2.089	4.039	0.5	Na	4.5
8	Na8	13.411	2.089	4.039	0.5	Na	4.5
9	Na9	11.26	9.83	-6.827	0.5	Na	4.5
10	Na10	1.141	7.721	6.827	0.5	Na	4.5
11	Na11	6.527	1.5	6.318	0.5	Na	4.5
12	Na12	5.873	16.051	-6.318	0.5	Na	4.5
13	Na13	0.709	10.275	4.951	0.5	Na	4.5
14	Na14	13.109	10.275	4.951	0.5	Na	4.5
15	Na15	-0.709	7.276	-4.951	0.5	Na	4.5
16	Na16	11.692	7.276	-4.951	0.5	Na	4.5
17	Na17	1.099	4.358	6.448	0.5	Na	4.5
18	Na18	11.302	13.193	-6.448	0.5	Na	4.5
19	Na19	6.137	13.133	4.821	0.5	Na	4.5
20	Na20	6.263	4.418	-4.821	0.5	Na	4.5
21	Na21	-1.878	12.522	2.021	0.5	Na	4.5
22	Na22	10.522	12.522	2.021	0.5	Na	4.5
23	Na23	1.878	5.028	-2.021	0.5	Na	4.5
24	Na24	14.279	5.028	-2.021	0.5	Na	4.5
25	O1	-0.823	9.725	-4.402	1	O	0.1
26	O2	11.578	9.725	-4.402	1	O	0.1
27	O3	0.823	7.826	4.402	1	O	0.1
28	O4	13.223	7.826	4.402	1	O	0.1
29	O5	10.652	4.725	2.626	1	O	0.1
30	O6	1.749	12.826	-2.626	1	O	0.1
31	O7	9.457	7.614	-3.751	1	O	0.1
32	O8	8.074	12.231	-0.841	1	O	0.1
33	O9	4.327	5.32	0.841	1	O	0.1
34	O10	10.868	11.089	3.886	1	O	0.1
35	O11	1.533	6.462	-3.886	1	O	0.1
36	O12	0.752	12.335	2.445	1	O	0.1

37	O13	8.739	16.55	-0.128	1	O	0.1
38	O14	6.196	16.463	2.067	1	O	0.1
39	O15	6.205	1.088	-2.067	1	O	0.1
40	O16	2.502	9.681	-0.453	1	O	0.1
41	O17	9.899	7.87	0.453	1	O	0.1
42	O18	2.37	9.012	-5.874	1	O	0.1
43	O19	10.03	8.539	5.874	1	O	0.1
44	O20	5.961	12.97	2.227	1	O	0.1
45	O21	6.439	4.581	-2.227	1	O	0.1
46	O22	3.609	11.19	-2.628	1	O	0.1
47	O23	8.792	6.36	2.628	1	O	0.1
48	O24	0.047	7.8	-6.516	1	O	0.1
49	O25	-0.047	9.751	6.516	1	O	0.1
50	O26	12.353	9.751	6.516	1	O	0.1
51	O27	5.117	16.575	-4.753	1	O	0.1
52	O28	7.283	0.976	4.753	1	O	0.1
53	O29	3.99	13.993	-2.344	1	O	0.1
54	O30	8.411	3.558	2.344	1	O	0.1
55	O31	2.689	5.586	-6.356	1	O	0.1
56	O32	9.711	11.964	6.356	1	O	0.1
57	O33	2.475	14.362	-4.914	1	O	0.1
58	O34	9.926	3.189	4.914	1	O	0.1
59	O35	-1.156	13.584	-0.183	1	O	0.1
60	O36	11.244	13.584	-0.183	1	O	0.1
61	O37	1.156	3.967	0.183	1	O	0.1
62	O38	5.373	16.263	-1.334	1	O	0.1
63	O39	7.027	1.288	1.334	1	O	0.1
64	O40	7.151	10.829	1.179	1	O	0.1
65	O41	5.25	6.722	-1.179	1	O	0.1
66	O42	12.229	2.41	6.137	1	O	0.1
67	O43	7.407	11.185	5.132	1	O	0.1
68	O44	4.993	6.366	-5.132	1	O	0.1
69	O45	10.313	10.66	0.739	1	O	0.1
70	O46	2.087	6.89	-0.739	1	O	0.1
71	O47	14.488	6.89	-0.739	1	O	0.1
72	O48	2.749	2.887	5.745	1	O	0.1
73	O49	7.913	5.888	-5.524	1	O	0.1
74	O50	6.41	14.058	-2.759	1	O	0.1
75	O51	5.99	3.493	2.759	1	O	0.1
76	O52	1.294	14.918	-0.225	1	O	0.1
77	O53	9.591	14.866	-2.367	1	O	0.1

78	O54	5.335	9.36	-0.753	1	O	0.1
79	O55	7.066	8.191	0.753	1	O	0.1
80	O56	8.041	9.571	-1.355	1	O	0.1
81	O57	4.359	7.98	1.355	1	O	0.1
82	O58	6.945	14.225	-5.738	1	O	0.1
83	O59	5.455	3.326	5.738	1	O	0.1
84	O60	4.809	13.003	-4.596	1	O	0.1
85	O61	7.591	4.547	4.596	1	O	0.1
86	O62	8.306	12.63	-3.757	1	O	0.1
87	O63	4.094	4.921	3.757	1	O	0.1
88	O64	1.975	16.042	2.265	1	O	0.1
89	O65	10.425	1.509	-2.265	1	O	0.1
90	O66	0.131	9.344	1.636	1	O	0.1
91	O67	12.531	9.344	1.636	1	O	0.1
92	O68	-0.131	8.207	-1.636	1	O	0.1
93	O69	12.27	8.207	-1.636	1	O	0.1
94	O70	8.549	13.114	1.965	1	O	0.1
95	O71	3.852	4.437	-1.965	1	O	0.1
96	O72	9.437	8.407	-7.361	1	O	0.1
97	O73	2.963	9.144	7.361	1	O	0.1
98	O74	8.128	17.182	-3.908	1	O	0.1
99	O75	1.118	3.089	-3.496	1	O	0.1
100	O76	3.415	13.121	2.831	1	O	0.1
101	O77	8.986	4.43	-2.831	1	O	0.1
102	O78	4.71	9.962	-4.947	1	O	0.1
103	O79	7.69	7.589	4.947	1	O	0.1
104	O80	2.649	11.601	-5.096	1	O	0.1
105	O81	9.751	5.95	5.096	1	O	0.1
106	O82	8.297	10.036	-4.192	1	O	0.1
107	O83	4.103	7.515	4.192	1	O	0.1
108	O84	8.993	9.116	2.998	1	O	0.1
109	O85	3.407	8.435	-2.998	1	O	0.1
110	O86	6.802	8.833	-6.293	1	O	0.1
111	O87	5.598	8.718	6.293	1	O	0.1
112	O88	10.119	11.143	-2.328	1	O	0.1
113	O89	2.281	6.408	2.328	1	O	0.1
114	O90	6.021	9.095	3.365	1	O	0.1
115	O91	6.38	8.456	-3.365	1	O	0.1
116	O92	6.677	11.533	-5.777	1	O	0.1
117	O93	5.723	6.018	5.777	1	O	0.1
118	O94	5.512	12.512	-0.417	1	O	0.1

119	O95	6.888	5.039	0.417	1	O	0.1
120	O96	6.465	11.224	-3.106	1	O	0.1
121	O97	5.935	6.327	3.106	1	O	0.1
122	O98	2.862	12.28	-0.016	1	O	0.1
123	O99	9.539	5.271	0.016	1	O	0.1
124	O100	4.407	14.495	0.509	1	O	0.1
125	O101	7.994	3.056	-0.509	1	O	0.1
126	O102	4.497	10.892	1.582	1	O	0.1
127	O103	7.904	6.659	-1.582	1	O	0.1
128	O104	2.753	6.083	6.521	1	O	0.1
129	O105	9.647	11.468	-6.521	1	O	0.1
130	O106	0.433	11.347	-0.728	1	O	0.1
131	O107	12.833	11.347	-0.728	1	O	0.1
132	O108	11.968	6.204	0.728	1	O	0.1
133	O109	1.49	10.204	-3.052	1	O	0.1
134	O110	10.91	7.347	3.052	1	O	0.1
135	O111	6.905	14.402	0.063	1	O	0.1
136	O112	5.495	3.149	-0.063	1	O	0.1
137	O113	4.687	2.849	-4.411	1	O	0.1
138	O114	-0.739	13.139	-3.881	1	O	0.1
139	O115	11.662	13.139	-3.881	1	O	0.1
140	O116	0.739	4.412	3.881	1	O	0.1
141	O117	13.139	4.412	3.881	1	O	0.1
142	W1	3.176	9.699	-4.062	1	W	0.9
143	W2	9.224	7.852	4.062	1	W	0.9
144	W3	7.406	12.47	0.669	1	W	0.9
145	W4	4.994	5.08	-0.669	1	W	0.9
146	W5	6.697	9.702	-4.852	1	W	0.9
147	W6	5.704	7.849	4.852	1	W	0.9
148	W7	3.016	12.963	-3.819	1	W	0.9
149	W8	9.384	4.588	3.819	1	W	0.9
150	W9	8.468	11.049	-2.389	1	W	0.9
151	W10	3.932	6.502	2.389	1	W	0.9
152	W11	6.773	12.999	-4.485	1	W	0.9
153	W12	5.627	4.552	4.485	1	W	0.9
154	W13	5.421	14.452	-1.287	1	W	0.9
155	W14	6.98	3.099	1.287	1	W	0.9
156	W15	5.626	9.583	1.608	1	W	0.9
157	W16	6.775	7.968	-1.608	1	W	0.9
158	W17	3.987	12.629	1.127	1	W	0.9
159	W18	8.413	4.922	-1.127	1	W	0.9

160	W19	1.878	10.986	-1.372	1	W	0.9
161	W20	10.522	6.565	1.372	1	W	0.9
162	Zn1	5.17	11.151	-1.761	1	Zn	0.04
163	Zn2	7.23	6.4	1.761	1	Zn	0.04
164	Zn3	8.9	9.581	0.795	0.5	Zn	0.04
165	Zn4	3.501	7.97	-0.795	0.5	Zn	0.04
166	W21	8.9	9.581	0.795	0.5	W	0.9
167	W22	3.501	7.97	-0.795	0.5	W	0.9

Alternative models of the precursor structure using PDFgui

Keggin cluster with fixed relative positions.

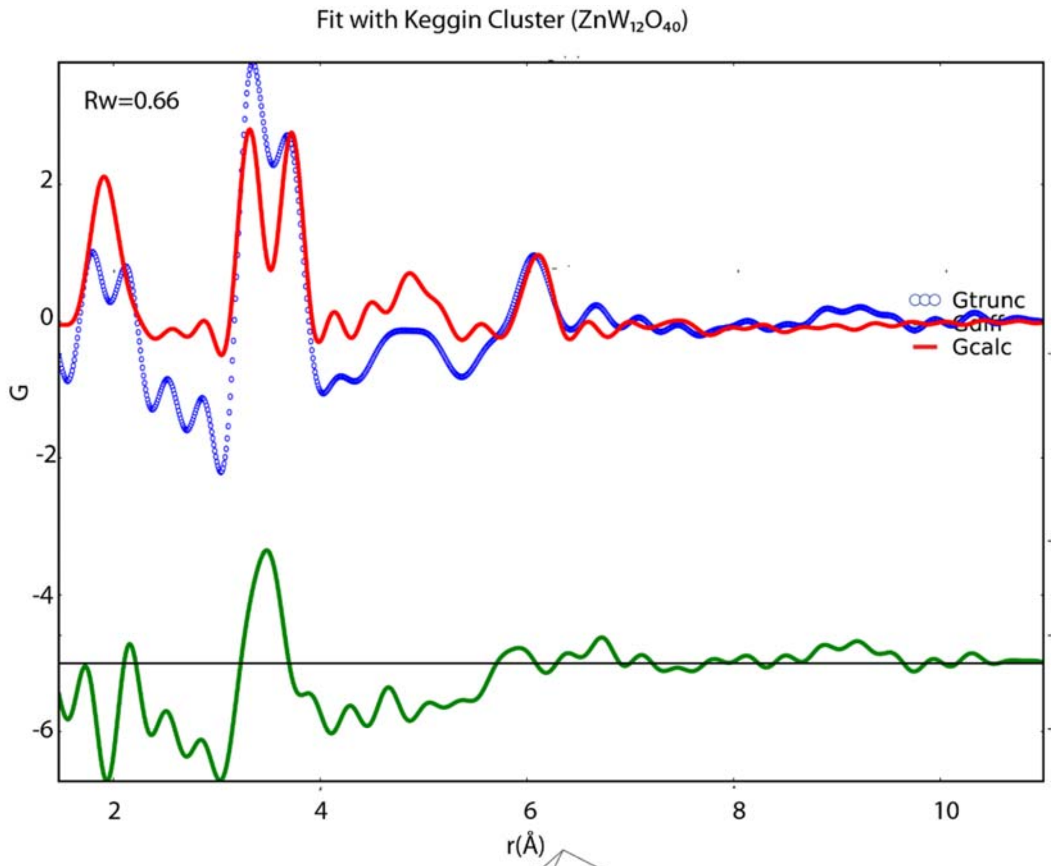

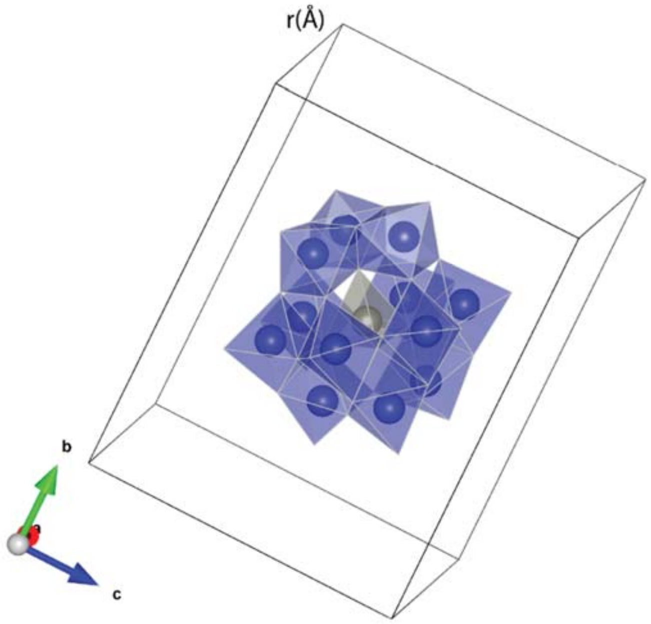

Modified Keggin cluster not refining positions but with correct synthesis stoichiometry, that is: Zn:W 1:1

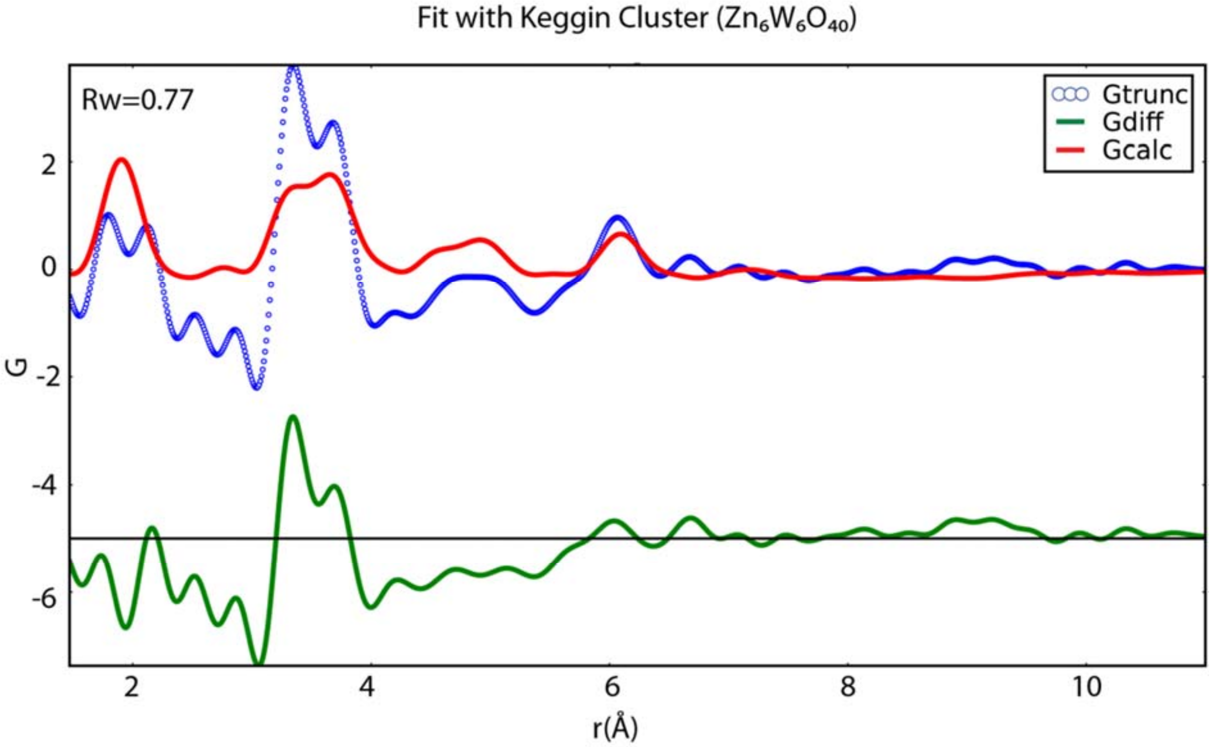

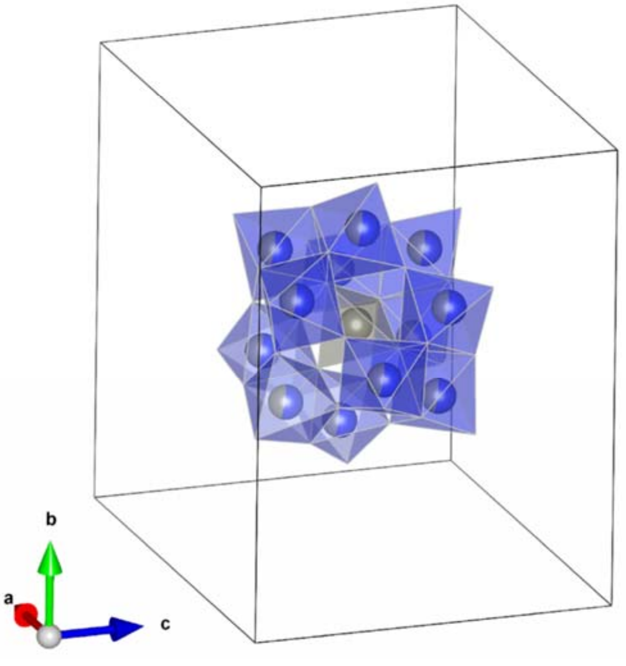

Keggin cluster with 1:1 Stoichometry refining all positions.

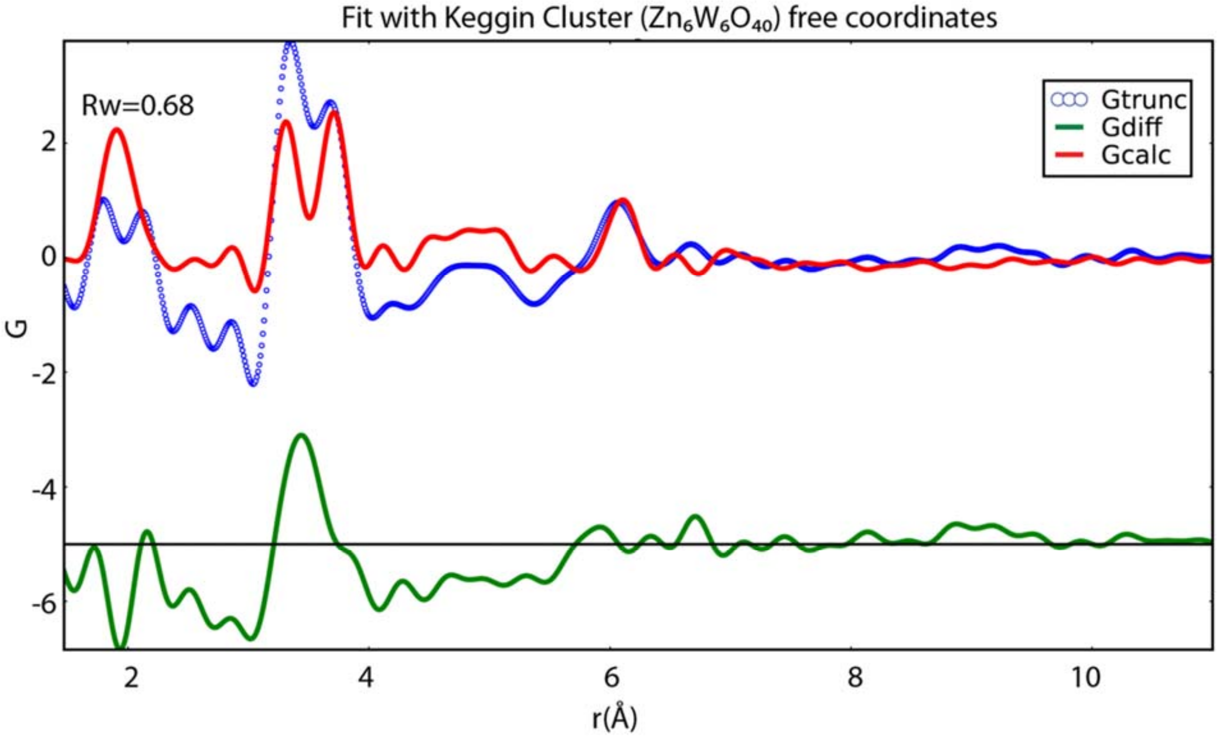

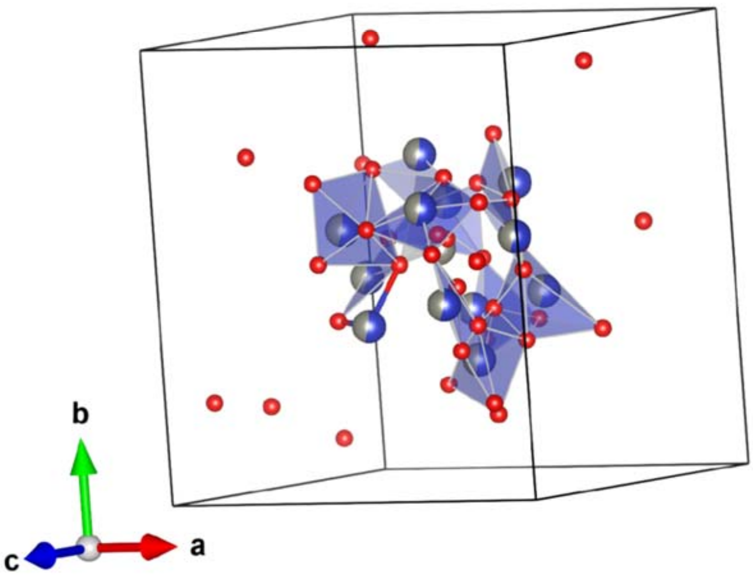

Keggin cluster with ZnO

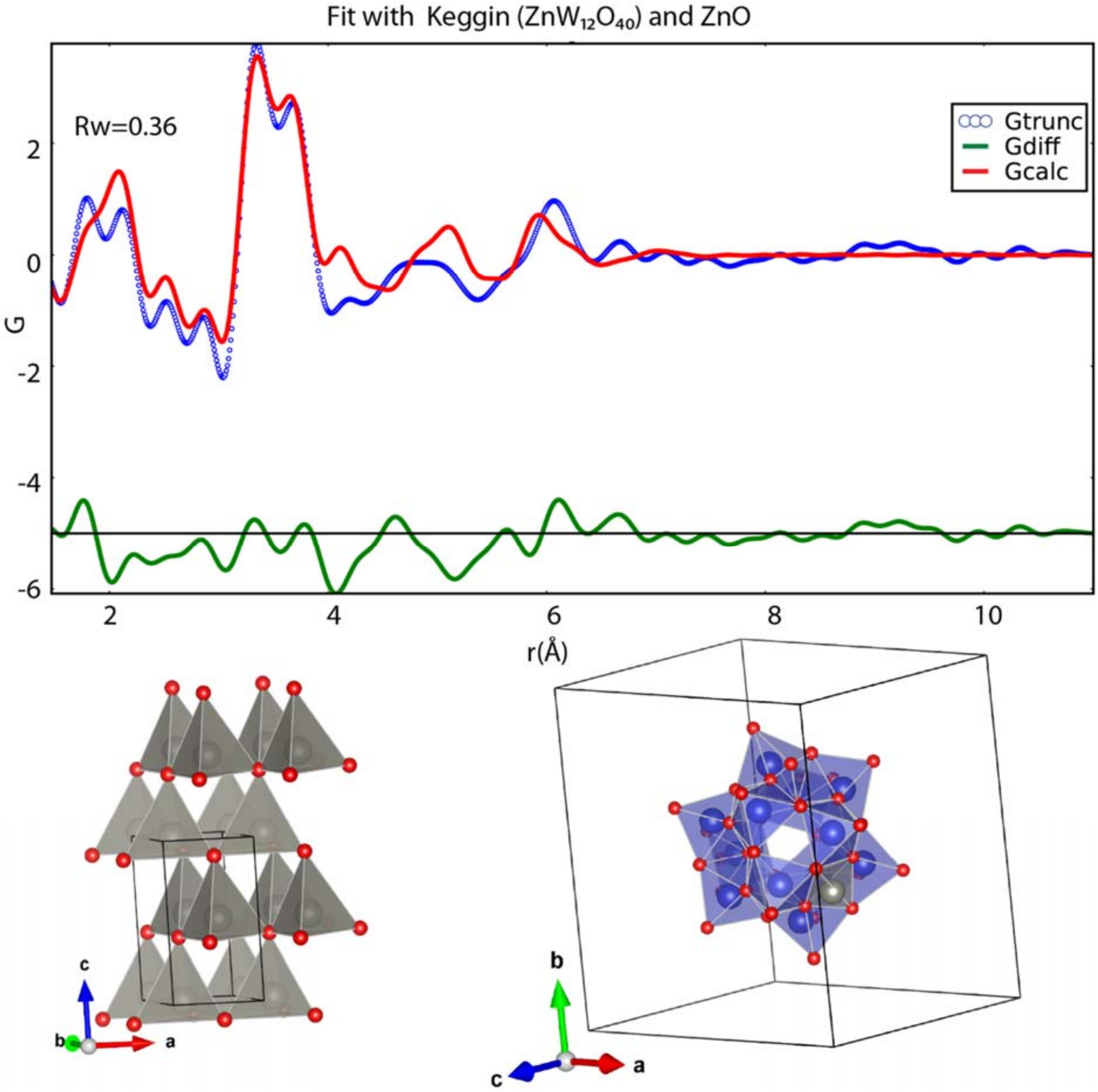

Keggin Cluster + ZnO refining positions

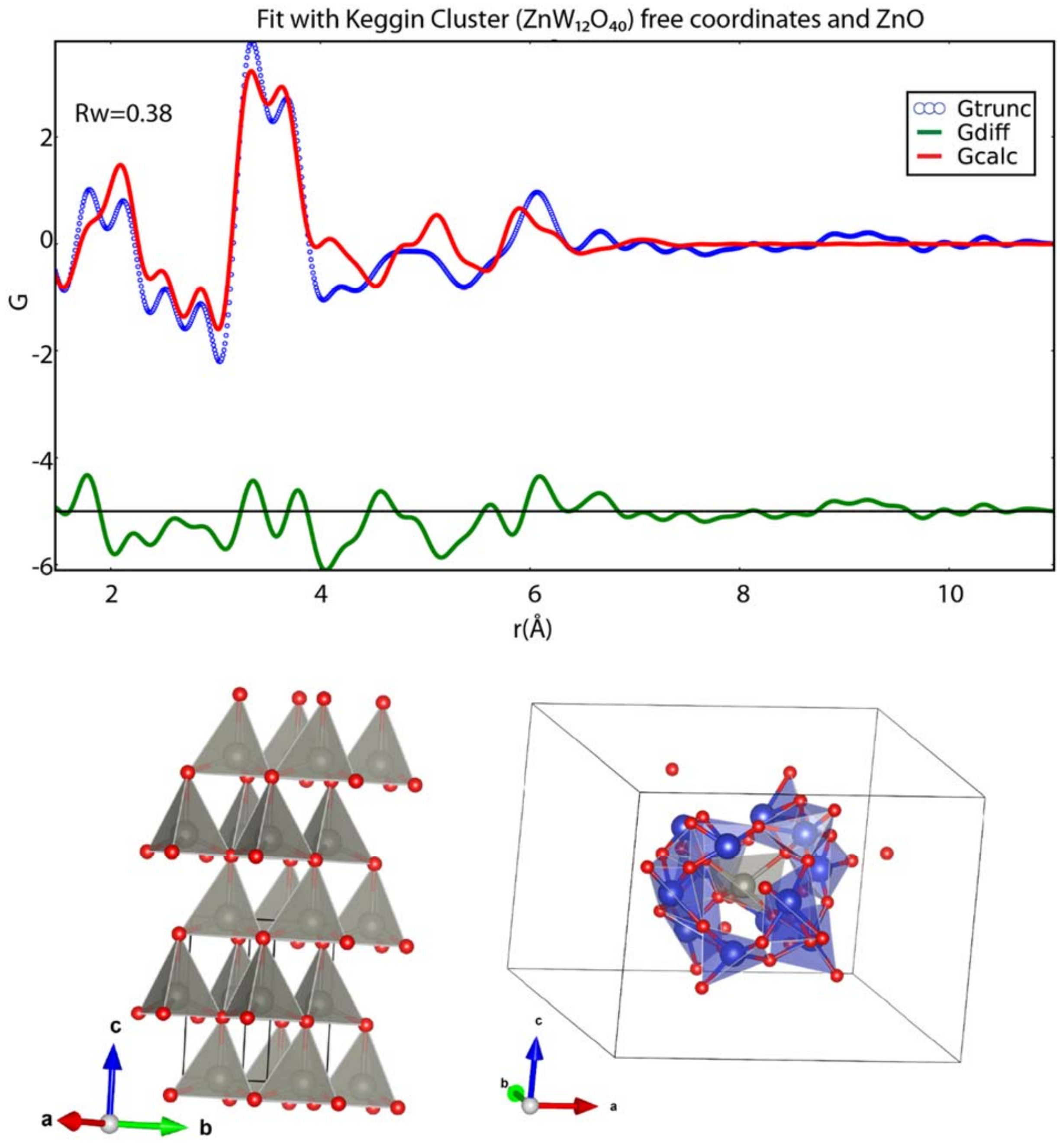

Simulations using Debye Scattering function of various possible precursor structures

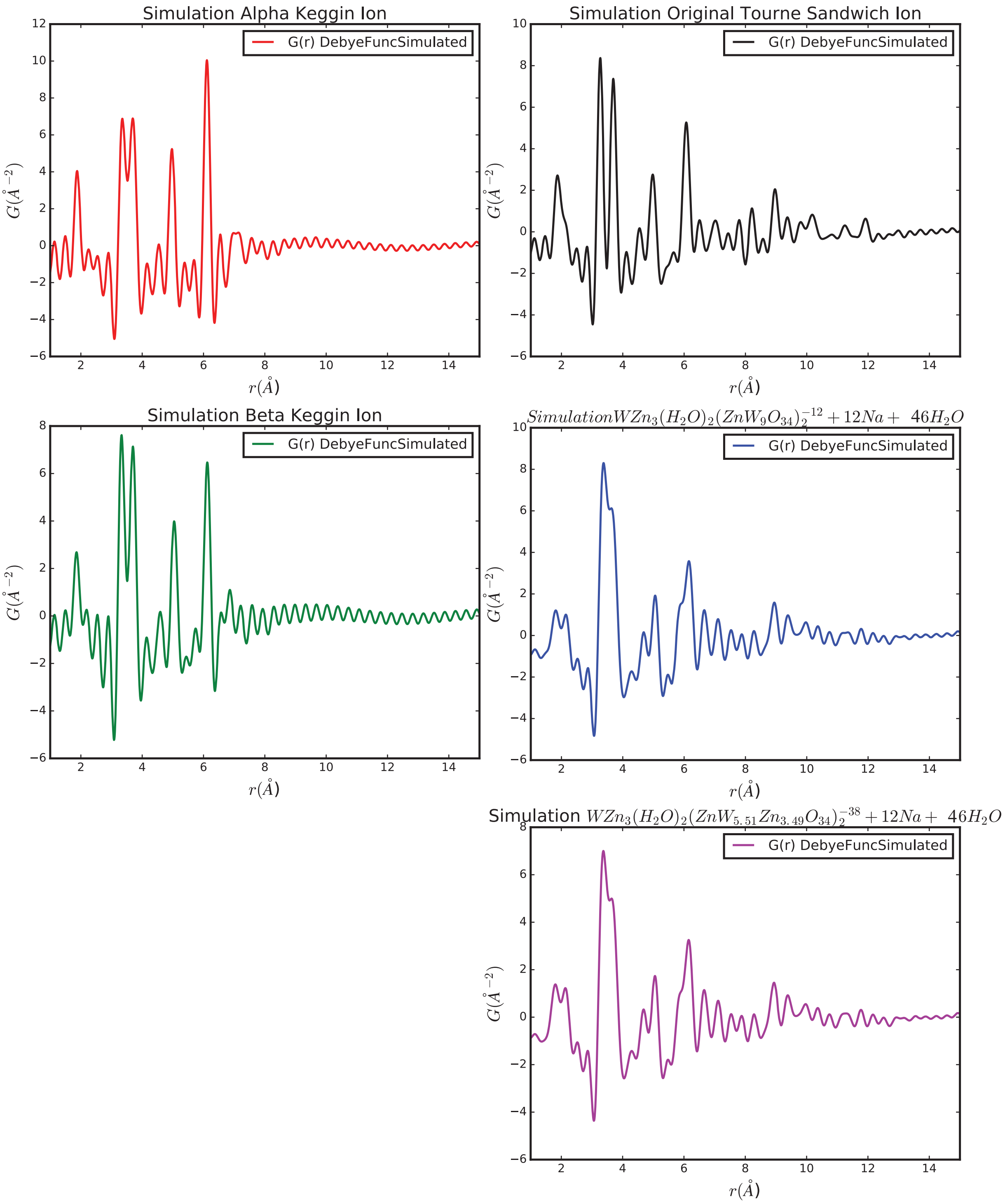

References

1. M. Jarvinen, *J. Appl. Crystallogr.*, 1993, **26**, 525-531.
2. N. Popa, *J. Appl. Crystallogr.*, 1998, **31**, 176-180.
3. C. Tyrsted, J. Becker, P. Hald, M. Bremholm, J. S. Pedersen, J. Chevallier, Y. Cerenius, S. B. Iversen and B. B. Iversen, *Chem. Mater.*, 2010, **22**, 1814-1820.
4. B. E. Warren, *X-ray diffraction*, Dover Publications, New York, Dover edn., 1990.
5. P. Thompson, D. E. Cox and J. B. Hastings, *J. Appl. Crystallogr.*, 1987, **20**, 79-83.
6. A. R. Stokes and A. J. C. Wilson, *Proc. Phys. Soc., London*, 1944, **56**, 174-181.
7. D. M. Trots, A. Senyshyn, L. Vasylechko, R. Niewa, T. Vad, V. B. Mikhailik and H. Kraus, *J. Phys.: Condens. Matter*, 2009, **21**, 325402.
8. C. L. Farrow, P. Juhas, J. W. Liu, D. Bryndin, E. S. Bozin, J. Bloch, T. Proffen and S. J. L. Billinge, *J. Phys.: Condens. Matter*, 2007, **19**.
9. A.-C. Dippel, H.-P. Liermann, J. T. Delitz, P. Walter, H. Schulte-Schrepping, O. H. Seeck and H. Franz, *J. Synchrotron Radiat.*, 2015, **22**.
10. R. Lopez and R. Gomez, *J. Sol-Gel Sci. Technol.*, 2012, **61**, 1-7.
11. M. L. Myrick, M. N. Simcock, M. Baranowski, H. Brooke, S. L. Morgan and J. N. McCutcheon, *Appl. Spectrosc. Rev.*, 2011, **46**, 140-165.
12. S. P. Tandon and J. P. Gupta, *Phys. Status Solidi*, 1970, **38**, 363-&.
13. C. A. Schneider, W. S. Rasband and K. W. Eliceiri, *Nat. Methods*, 2012, **9**, 671-675.
